# Bacterial diversity, quantitative risk assessment of spring waterborne pathogens and their biocontrol through phyto-biosorbents

**DOI:** 10.1371/journal.pone.0349096

**Published:** 2026-07-13

**Authors:** Aysha Aziz, Saiqa Andleeb, Nuzhat Shafi, Wajid Arhsad Abbasi, Aisha Nazir

**Affiliations:** 1 Fisheries Laboratory, Department of Zoology, University of Azad Jammu and Kashmir, King Abdullah Campus, Muzaffarabad, Pakistan; 2 Microbial Biotechnology and Vermitechnology Laboratory, Department of Zoology, University of Azad Jammu and Kashmir, King Abdullah Campus, Muzaffarabad, Pakistan; 3 Computational Biology and Data Analysis Laboratory, Department of Computer Science and Information Technology, University of Azad Jammu and Kashmir, King Abdullah Campus, Muzaffarabad, Pakistan; 4 Institute of Botany, University of Punjab, Quaid-e-Azam Campus, Lahore, Pakistan; University of Jeddah, SAUDI ARABIA

## Abstract

Bacterial contamination of drinking water is a significant cause of water-borne illnesses in many developing countries, where water sources are commonly shared by the community. Therefore, the recent research was designed to screen the bacterial diversity associated with spring water, to assess the bacterial risk in both adults and children, and to remediate bacterial contaminants using green remediation technology via *Brassica rapa* and *Spinacia oleracea*. Microbial growth media, microscopic techniques, biochemical tests, ribotyping, antibiogram, and resistogram analysis were used to characterize bacterial isolates. The bactericidal effect of extracts of phytoabsorbents was evaluated against spring water-associated bacteria through the agar well diffusion method. The ex-situ remediation via phytoabsorbents was done to decontaminate spring water. *Bacillus amyloliquefaciens*, *Bacillus subtilis, Bacillus cereus*, *Bacillus anthracis, Lysinibacillus fusiformis*, *Bacillus wiedmannii*, *uncultured Bacillus sp.*, *Bacillus weihenstephanensis*, and *Bacillus thuringiensis* were characterized through the Maximum Likelihood method and Tamura-Nei model. The highest mean risk of illness (%) due to Bacillus species was recorded in children (56.67% to 70.0% compared to the adults (47.0% to 50.0%). All isolated spring water-associated bacteria were resistant to amoxycillin, aztreonam, tobramycin, tazobactam, ceftriaxone, and cefuroxime sodium. Similarly, all spring water-associated bacteria showed resistance against lead, cadmium, and chromium. The root extract of *B. rapa* and *S. oleracea* showed the maximum zone of inhibition of spring water-associated bacteria (10.0 ± 0.0 mm to 19.0 ± 0.0 mm) compared to the seeds and aerial parts extracts. Ex-situ remediation findings illustrated that both plants efficiently declined the microbial load, did not affect the sprouting and growth of *B. rapa* compared to the *S. oleracea,* and could be used as biosorbent to decontaminate spring water.

## 1. Introduction

Water is an invaluable natural resource and essential for all living things’ survival [1]. Since clean water is necessary for drinking, cleanliness, and food production, it is crucial for health, economic growth, and general well-being. By controlling body temperature, it maintains bodily functions. A robust economy depends on having access to clean water, which is acknowledged as a fundamental human right and supports sectors including tourism and agriculture [2,3]. Anthropogenic activities, i.e., agricultural, pharmaceutical, and industrial activities, produced contaminants in large quantities to contaminate water [4–6]. Similarly, hospital effluents, fecal matter, and cattle farms also increase the bacterial load in a water body [7,8].

Waterborne disease outbreaks such as typhoid, bloody diarrhea, Shigellosis, dysentery, cholera, watery diarrhea, and diarrhea caused by untreated wastewater discharge [9,10]. The existence of various fecal bacterial species such as *Clostridium perfringens, Streptococci* spp., *Salmonella typhi, Salmonella typhimurium, Salmonella enteritidis*, *Shigella* spp., *Proteus spp.,* and *Klebsiella* spp. in water bodies indicates the fecal contaminated water [11,12].

Bacterial contamination of drinking water poses serious threats to public health in developing nations [13]. Access to safe and clean water is a basic human right, yet the global water crisis continues to affect millions of lives. Currently, ensuring the availability and protecting the quality of freshwater resources worldwide remain among the most urgent environmental challenges [14,15]. Therefore, drinking water treatment is a high priority and various processes like filtration systems, oxidation processes, multi-barrier systems including ozonation, reverse osmosis, chlorination, iodine, membrane and nano-filtration, hydrogen peroxide, photocatalytic method, and UV light disinfection have been developed to eradicate the bacteria and heavy metals from the aqueous systems [16–20]. But these all technologies have some drawbacks, i.e., time-consuming, less effective against bacterial spores, environmental destructive technologies, operational and maintenance costs, inefficient, higher concentration required, change the taste, odor and color of water.

Phytoremediation technologies, i.e., phytoextraction, rhizofiltration, phytostabilization, and phytotransformation/phytodegradation, have been proposed as effective, eco-friendly, and low-cost technologies for the remediation of polluted water [21–24]. Various researchers reported the significant use of emergent, floating, or merged plant species to reduce pollutants from the target water bodies [21–24]. To remove, degrade, or immobilize pollutants, phytoremediation employs a variety of methods, such as (1) immobilization (phytostabilization); (2) degradation (rhizodegradation, phytodegradation); (3) accumulation (phytoextraction, rhizofiltration); and (4) dissipation (phytovolatilization) [25]. Previous literature also reported that phytoremediation is a promising technique for pharmaceutical remediation [25,26]. Several aquatic and terrestrial plants, i.e., *Eichhornia crassipes (water hyacinth), Azollapinnata, Pistia stratiotes, Salvinia molesta, S. polyrhiza, L. minor, P.* *australis, P. karka, T. dominguensis, Cyperus alternifolius, Lpomoea aquatica,* and *T. latifolia* have been successfully employed for wastewater remediation [27–33].

Even though *Brassica rapa* and *Spinacia oleracea* have been explored for removing heavy metals from various contaminated soil [34–37]. Beside this, the potential of *Brassica rapa* and *Spinacia oleracea* as natural biosorbents remains largely under-investigated. Therefore, the current research aimed to remove bacterial contamination from spring water through root-merged technology via wetland system using *B. rapa* and *S. oleracea*. *Brassica rapa* and *Spinacia oleracea* are cultivated in Azad Jammu and Kashmir, Pakistan, due to their nutritional worth and potential benefits to human health. Furthermore, no comprehensive studies have evaluated their ability to remove multiple contaminants simultaneously or their applicability in real spring-water matrices rather than synthetic laboratory solutions. This gap highlights the need for systematic research to determine the feasibility, mechanisms, and effectiveness of *B. rapa* and *S. oleracea* as cost-effective, eco-friendly biosorbents for spring-water decontamination. The findings from this research may offer a promising tool for promoting environmental sustainability.

## 2. Materials and methods

### 2.1. Collection of spring water, garden soil, and vermicompost

A sampling of spring water was conducted during morning time from 30 spring locations of Naluchi, Nisar Camp, Chella, Lower plate, Tariqabad, Domel, Chattar, and Ambor of the districts Muzaffarabad, Azad Jammu and Kashmir (AJ&K), Pakistan, during May 2022 in sterilized plastic bottles. Hundred milliliters of spring water (in triplicate) was brought into Microbial Biotechnology/Vermitechnology Laboratory, Department of Zoology, King Abdullah Chattar Kalas Campus, University of Azad Jammu and Kashmir (UAJ&K), Muzaffarabad, Pakistan, placed at room temperature, and investigated instantly. These spring water samples are used for drinking by the local population. Muzaffarabad, situated in Pakistan’s northeast between latitudes 34.24° and longitudes 73.22°, is the capital of Azad Jammu & Kashmir, and spans 2496 square kilometers. The weather in this area is highland and subtropical. Temperatures range from 42°C to −3°C, and the average annual rainfall is between 1000 and 1300 mm. The landscape of Muzaffarabad is rugged and mountainous. The garden soil and vermicompost were collected from the University of Azad Jammu and Kashmir (UAJ&K), Muzaffarabad, and used for the growth of phytobiosorbents.

### 2.2. Bacterial diversity analysis

Various aseptic techniques (spreading and streaking) and morphological studies were used for the isolation and identification of spring water-associated bacteria, like staining, biochemical tests, microbial differential and selective media, antibiogram (sensitivity towards antibiotics), resistogram (sensitivity towards heavy metals), and molecular analysis (ribotyping) were carried out.

#### 2.2.1. Isolation of bacteria.

A 100 ml of spring water was collected and placed at room temperature for bacterial isolation. The serial dilution method, as described by Somasegaran and Hoben [38], was applied to isolate bacteria associated with spring water samples. Nutrient broth medium (NBM) was used for bacterial growth. Serial dilutions of 10 ⁻ ² to 10 ⁻ ⁵ were prepared and incubated at room temperature for 24 h to obtain single bacterial colonies. After incubation, the sample (10 µl) was spread onto nutrient agar medium (NAM) and incubated overnight at 37°C. The next day, multiple bacterial colonies were observed in the 10 ⁻ ², 10 ⁻ ³, 10 ⁻ ⁴, and 10 ⁻ ⁵ dilutions. The bacterial load (colony-forming unit) in the original sample was calculated using the formula: CFU = (Number of colonies × Dilution factor)/ Volume of inoculum. A total of 47 spring water-associated bacteria (SWAB) were selected, grown in NBM, and incubated at 37°C for 24 h. The isolates were then purified through sub-culturing via NBM and stored at −20°C in 60% glycerol.

#### 2.2.2. Pre-characterization of bacteria.

Morphological characteristics such as shape, size, and type of colony were observed through microscopic/ staining techniques (Gram staining and Endospore staining) according to the APHA (2022) protocol [39–42], and using nutritional media (nutrient agar medium; NAM, tryptone soya agar; TSA, mannitol salt agar; MSA, and MacConkey agar; MA, eosin methylene blue agar; EMB, Thiosulfate Citrate Bile Salts Sucrose; TCBS}. All nutritional media were prepared and autoclaved at 121 °C for 20 min before use. MacFaddin [43] method was used to test the production of catalase in bacterial isolates (2A-30D). After being streaked on nutrient agar and cultured for 24 hours at 37°C, the isolates were moved to a glass slide. Catalase activity was detected by dropwise addition of 3% H_2_O_2_; the development of bubbles within 10 seconds showed positivity, while the absence of bubbles indicated negativity.

Jurtshuk and McQuitty, [44] approach was used to assess the oxidase activity of bacterial isolates. After 24 hours of growth at 37°C on nutritional agar, 1% oxidase reagent was added to the isolates. The presence of the oxidase enzyme was shown by the emergence of a purple color. For the coagulase test, a few colonies of the test organism were combined with 0.5 mL of plasma on a sterile glass plate. After gently rotating the mixture for ten seconds, the formation of a clot was monitored for ten to thirty seconds. The development of a clot verified the existence of coagulase-positive results [45].

In the KOH test, a loopful of bacterial growth on a microscope slide was combined with two drops of 3% KOH. Gram-negative status was confirmed by the quick development of stringy, viscous material within 30 seconds of mixing [41,46]. In the IAA test, the isolates were cultivated for seven days at 28 ± 2°C in Luria Bertani (LB) broth medium containing 100 mg/L tryptophan, a precursor of IAA. Each test tube was filled with Kovac’s reagent after incubation, and the mixture was then incubated for an additional ten to twenty minutes. Positive IAA generation was thought to be indicated by the creation of a clear cherry red color ring at the medium’s surface [47]. For HCN production, King’s B medium with 4.4 g/l glycine was incubated at 28 ± 2°C for 24–48 hours. Following incubation, Whatman No. 1 filter paper dipped in 0.5 percent picric acid and 2 percent sodium carbonate was inserted in the lid of Petri dishes, and the bacterial isolates were streaked on the King’s B medium. The dishes were then incubated at 28 ± 2°C for 48–72 hours. The filter paper’s color changed from bright yellow to orange-brown, indicating a successful cyanide manufacturing outcome [48,49].

For the urease test, 2.95 g of urea powder was dissolved in 150 ml of distilled water to make urea broth, which was then autoclaved. After that, a wire loop was used to aseptically inoculate the bacterial sample. A few drops of phenol red indicator were added to the culture following a 24-hour incubation period at 37°C. Positive urease activity was represented by pink, whilst negative urease activity was indicated by orange [43]. In lipolytic activity, bacterial isolates were cultivated for 24 hours at 35 ± 2°C on nutritional agar plates supplemented with 1% Tween 80. After incubation, the formation of a clear, opaque zone around the colonies was thought to be a sign of lipolytic activity [50]. According to Ashwini et al. [51], bacterial strains were cultivated on amylase medium at 35°C for 48 hours to examine amylase production. Iodine solution was applied dropwise to the bacterial colonies after incubation. The existence of amylase activity was shown by the formation of a clear zone around the colonies. According to Linares-Morales et al. [52], bacterial isolates were cultivated on skimmed milk agar medium in order to examine proteolytic activity. For a whole day, the isolates were incubated at 35 ± 2°C. The presence of proteolytic enzymes was confirmed when a clear zone appeared around the isolates after incubation, indicating casein breakdown.

#### 2.2.3. Antibiogram analysis.

Antibiotic analysis was performed using the Kirby-Bauer disk diffusion method (CSLI, 2022) [53]. Ten Oxoid™ Blank Antimicrobial Susceptibility discs such as Amoxycillin (30 μg), Aztreonam (30 μg), Tobramycin (10 μg), Pipemidic acid (20 μg), Tazobactam (10 μg), Cefotaxime (30 μg), Cefuroxime sodium (30 μg), Ceftriaxone (30 μg), Gentamicin (10 μg), and Enrofloxacin (10 μg) were used in the current study. Bacterial cultures were grown in Oxoid nutrient broth (NBM; CM1) for 24 h at 37°C, then 1.5 × 10⁸ CFU/mL mixed with nutrient agar medium (NAM; CM003) and poured into sterilized Petri dishes. Antibiotic discs were applied, and plates were incubated for 24 h 37°C. Inhibition zone diameters (IZD) were measured after 24–48 h [54]. The results of the disk diffusion test will be qualitative and help in categorizing as (i.e., susceptible, intermediate, or resistant). The zone diameters were measured, and results were interpreted according to Clinical and Laboratory Standards Institute (CLSI) guidelines [55]. Zones ≥ 10 mm indicated susceptibility, while zones ≤ 10 mm indicated resistance.

#### 2.2.4 Resistogram analysis.

Lead (Pb) nitrate, cadmium (Cd) nitrate, and chromium (Cr) nitrate were prepared for resistogram analysis and were done using the agar well diffusion method [56]. Metal resistance testing was performed on bacterial isolates from spring water samples to evaluate their adaptive tolerance to heavy metal contamination, which may indicate environmental pollution levels and potential co-selection for antimicrobial resistance. Nutrient agar (oxide: CMOO3) and nutrient broth media (Oxide: CM1) were used for bacterial growth. The bacteria (1.5 × 10⁸ CFU/mL) were added to a nutrient broth medium for growth and incubated for 24 h on a rotary shaker at 37°C. The incubated culture was mixed in a freshly prepared nutrient agar medium (NAM) at 45°C. The mixture was poured into sterilized Petri dishes and solidified in a laminar flow at room temperature. Three wells (5 mm in diameter) in each plate were made by using a sterilized micropipette tip. In each prepared well about 30 µl of HMs (100 µg/mL of dH_2_O) was added and then placed for 24 h at 37ºC. Inhibition zone diameters (IZD) were measured after 24–48 h [55], and results were categorized as susceptible, intermediate, or resistant based on zone size [55]. The zone diameters were interpreted according to CLSI guidelines [55]. Zones ≥ 10 mm indicated susceptibility, while zones ≤ 10 mm indicated resistance.

#### 2.2.5. Molecular analysis.

Genomic DNA was isolated from samples using the QIAGEN DNeasy Mini Kit, utilizing the spin column method as per the standard protocol [57] and quantified through a spectrophotometer at A260/A280. The PCR Primers, such as 27F 5’ (AGA GTT TGA TCM TGG CTC AG) 3’, and PCR Primer: 1492R 5’ (TAC GGY TAC CTT GTT ACG ACT T) 3’ were used for ribotyping. The PCR conditions were initial denaturation (94 °C for 5 min), denaturation (94 °C for 30 sec), annealing (52.7 °C for 35 sec), extension (72 °C for 2 min), final extension (72 °C for 5 min), and 35 cycles, and the expected product size was 1.4 kb to 1.6 Kb. After PCR analysis, 19 samples were sent to Macrogen company, Korea, for sequence analysis, and 16S rRNA sequencing Primers, i.e., 785F 5’ (GGA TTA GAT ACC CTG GTA) 3’ and 907R 5’ (CCG TCA ATT CMT TTR AGT TT) 3’ were used. For sequence analysis, the following software and databases were utilized: National Center for Biotechnology Information (NCBI) and Basic Local Alignment Search Tool (BLAST), and Molecular Evolutionary Genetics Analysis (MEGAX). These tools were employed to determine the evolutionary relationship with other species using Distance Tree analysis (Fast Minimum Evolution Method, with a maximum sequence difference of 0.75) [58].

### 2.3. Quantitative microbial risk assessment

The risk to human health posed by adults and children being exposed to harmful bacteria after drinking spring water was evaluated using QMRA analysis. The quantitative data on human pathogen exposures (exposure assessment) and the likelihood that exposures would cause infection or sickness (dose-response relationship) are combined in QMRA [59,60]. Hazard identification (1), dose-response assessment (2), exposure assessment (3), and risk characterization (4) were the stages followed in the QMRA. The hazard identification process found the infections and the negative health impacts. The dose-response assessment was established based on both the exponential model (P_inf =_1 − e^−r⋅d^) and the Beta-Poisson model (P_inf_ = 1−(1 + d/β)^−α^), where P_inf_ = Probability of infection per day; r = Pathogen-specific infectivity constant (0.000001); d = Dose (e.g., CFU/person/day); α\alpha, β\beta = Pathogen-specific Beta-Poisson model parameters. As a component of QMRA, both models establish the relationship between the dose of a pathogen and the probability of adverse health effects such as infection, illness, or death occurring in the exposed population [61,62]. The standard α and β values are not well established for bacillus species, but in QMRA modeling studies, surrogate values are used based on experimental data or assumed distributions such as (0.27 and 100000 for adults) and (0.41 and 70000 for children). The annual risk of infection (P_inf.annual_) and the probability of illness per infection (P_ill_) means the chance that a person will develop a disease after being exposed to a harmful agent such as a pathogen, toxin, contaminated food, or unsafe environment were also calculated using (P_inf.annual_)=1-(1-P_inf_)^n^ and Pill = P_inf.annual_ x P_ill/inf_. Risk interpretation was carried out as Negligible risk (< 10 ⁻ ⁶/0.000001), Very low risk (10 ⁻ ⁶ – 10 ⁻ ⁵), generally acceptable (10 ⁻ ⁵ – 10 ⁻ ⁴), Low risk (> 10 ⁻ ⁴), may be acceptable with justification, Exceeds WHO health targets → intervention required, and High risk (> 10 ⁻ ²/1%) – unacceptable.

### 2.4. In vitro antibacterial effect of hyperaccumulators

#### 2.4.1. Extraction of Plant Materials.

*Spinacia oleracea* and *Brassica rapa* plants and seeds were purchased from the market of Muzaffarabad, AJ&K, Pakistan. Whole plants were washed with running tap water to remove dust or sand, and dried with sterilized filter paper. The aerial parts (leaves and stems) and roots were separated, weighed (20 g each), and macerated using a mortar and pestle. The crude aqueous extracts were collected, filtered using Whatman No. 1 filter paper with an approximate pore size of 11 µm, and concentrated using a rotary evaporator at 40–45°C under reduced pressure for approximately 30–60 minutes. On the other hand, seeds of *Spinacia oleracea* and *Brassica rapa* were ground along with distilled water (20g/50 ml), crude extract were filtered and concentrated through rotary evaporator as mentioned above. After evaporation, 4 g of crude extract was dissolved in DMSO (1 ml) and used for antibacterial activity.

#### 2.4.2. Preparation of Microbial Cultures.

Nutrient broth and nutrient agar medium are used for the growth of spring water-associated bacteria (SWAB). All isolated bacteria (SWAB1 to SWAB19) were grown in a nutrient broth medium and incubated at 37°C for 24 h. The antibacterial activity of extracts of both *Brassica rapa* and *Spinach oleracea* was evaluated against spring water-associated bacteria.

#### 2.4.3. Agar Well Diffusion Method.

The agar well diffusion method was employed to assess the antibacterial activity of plant extracts [56]. Instead of heavy metals, 30 µl of aerial parts, roots, and seeds extracts were added. The protocol is the same as mentioned in Section 2.2.4.

### 2.5. Ex vivo spring water treatment

For the ex-situ phytoremediation of spring water, *B. rapa* and *S. oleracea* were used. Spring water samples such as CSW-1, CSW-20, CSW-21, CSW-22, CSW-23, CSW-24, CSW-25 and CSW-29 were selected for the remediation due to high CFU/mL thresholds. Garden soil was collected from the University grounds, Muzaffarabad, and all physiochemical parameters were analyzed before use. Small containers (capacity of 1.5 kg) having small holes were purchased, filled with soil, and approximately 60−70 seeds of *B. rapa* and *S. oleracea* were sown. After sowing, containers were placed in the trays having contaminated spring water (500 ml) and allowed to germinate for 20−30 days at 18 ± 2–23 ± 2 °C and 60–80% humidity for 10–12 hours/day. At the end of the experiment, water samples were collected, and microbial contamination (CFU/10 µL) was analyzed through streaking of treated spring water on nutrient agar plates. After incubation CFU/10 µL was counted through colony counter. The percentage reduction in bacterial growth was calculated as: (Number of colonies in control – Number of colonies in treatments/ Number of colonies in control) x 100. The effect of spring water on the growth of *B. rapa* and *S. oleracea* was monitored throughout the phytoremediation process. Seed germination and plant growth parameters such as plant length, root length, shoot length, and dry biomass were recorded from day 7 until the completion of the experiment. After experimentation, physicochemical parameters pH, temperature, electrical conductivity, turbidity, total dissolved solids, biological oxygen demand, chemical oxygen demand, and dissolved oxygen were measured to check the water quality. The mercury thermometer was used to measure the temperature, pH was measured using a calibrated pH meter (JENCO, Model: 6231N), EC and TDS were measured with a digital two-in-one conductivity + TDS meter with a cell constant of 1.0, turbidity was recorded using a turbid meter (NOVATECH Instrument, TU- 101), dissolved oxygen levels in the spring water were measured using an oximeter (Model: YK-22DOA), Biochemical oxygen demand (BOD) was determined using the titration method, specifically the Winkler procedure, and chemical oxygen demand (COD) of the sample spring waters was determined using the titration method.

### 2.6. Statistical analysis

All treatments were carried out in triplicate, and the findings were reported as a mean value with standard deviation (Mean ± SD) using the software Excel 16. All water samples were analyzed in triplicate. One-way analysis of variance (ANOVA) means were applied, and significant differences were applied as “a” indicates significant difference among seeds and all other treatment groups; “b” indicates significant difference among roots and all treatment groups, “c” indicates significant difference among aerial parts and all treatment groups. Statistical icons: single letter such a/b/c = p ≤ 0.05; double letter such as aa/bb/cc = p ≤ 0.01; triple letter such as aaa/bbb/ccc, = p ≤ 0.001.

## 3. Results

### 3.1. Morphological features

Results revealed that all bacterial isolates showed convex shape, round type, and medium-sized colonies (Supplementary Table 1). All SWABs were gram-positive rods and endospore-forming. Differential and selective cultural media showed that no growth was recorded on mannitol salt agar except SWAB-8 and SWAB-9. Yellow and pink colonies were observed on MacConkey agar, shiny pinkish colonies were seen on Eosin methylene blue medium, and mucoid green and yellow colonies on Thiosulfate citrate bile salts sucrose agar medium. On the other hand, SWAB-4, SWAB-8, SWAB-9, and SWAB-12 didn’t show growth on TCBS medium. Blood agar medium results revealed that isolated bacteria were β-hemolytic and α-hemolytic (Supplementary Table 1).

### 3.2. Biochemical characteristics

Results showed that all spring water-associated bacteria were oxidase and catalase-positive. SWAB-1, SWAB-2, SWAB-4, SWAB-5, SWAB-6, SWAB-7, SWAB-8, SWAB-11, SWAB-12, SWAB-14, and SWAB-16 showed coagulase positive. SWAB-2, SWAB-3, SWAB-4, SWAB-5, SWAB-10, SWAB-13, SWAB-14, SWAB-15, SWAB-17, and SWAB-19 produced indole acetic acid (Table 1). Similarly, all SWABs produced urease enzymes except SWAB-9, SWAB-10, SWAB-12, SWAB-14, and SWAB-17, lipase producers except SWAB-8, and SWAB-9, SWAB-1 and SWAB-1, SWAB-3, SWAB-4, SWAB-9, SWAB-10, SWAB-12, and SWAB-17 were not involved in amylase production. SWAB-7 produced HCN, SWAB-2 only produced ammonia, and SWAB-5 and SWAB-12 produced proteases (Table 1). It was observed that all isolated SWABs were KOH negative.

**Table 1:** Biochemical characterization of spring water-associated bacteria.

Bacterial IDs↓ Biochemical tests→	Oxid	Cata	Coag	KOH	IAA	Ure	NH_3_	HCN	Amy	Pro	Lip
**SWAB-1**	+	+	+	–	–	+	–	+	–	–	+
**SWAB-2**	+	+	+	–	+	+	+	–	+	–	+
**SWAB-3**	+	+	–	–	+	+	–	–	–	–	+
**SWAB-4**	+	+	+	–	+	+	–	–	–	–	+
**SWAB-5**	+	+	+	–	+	+	–	–	+	+	+
**SWAB-6**	+	+	+	–	–	+	–	–	+	–	+
**SWAB-7**	+	+	+	–	–	+	–	+	+	–	+
**SWAB-8**	+	+	+	–	–	+	–	–	+	–	+
**SWAB-9**	+	+	–	–	–	–	–	–	–	–	–
**SWAB-10**	+	+	–	–	+	–	–	–	–	–	+
**SWAB-11**	+	+	+	–	–	+	–	–	+	–	+
**SWAB-12**	+	+	+	–	–	–	–	–	–	+	+
**SWAB-13**	+	+	–	–	+	+	–	–	+	–	+
**SWAB-14**	+	+	+	–	+	–	–	–	+	–	+
**SWAB-15**	+	+	–	–	+	+	–	–	+	–	+
**SWAB-16**	+	+	+	–	–	+	–	–	+	–	+
**SWAB-17**	+	+	–	–	+	–	–	–	–	–	+
**SWAB-18**	+	+	–	–	–	+	–	–	+	–	+
**SWAB-19**	+	+	–	–	+	+	–	–	+	–	+

Spring water bacteria (SWAB), Oxidase (Oxid), Catalase (Cata), Coaglase (Coag), Potassium hydroxide (KOH), Indole acetic acid (IAA), Urease (Ure), Ammonia (NH_3_), Hydrogen cyanide (HCN), Amylase (amy), Protease (Pro), Lipase (Lip), + (Positive), – (Negative)

### 3.3. Antibiogram and resistogram analysis

Table 2 indicates that all isolated SWABs (SWAB-1 to SWAB-19) were resistant against Amoxycillin (AMC-30), Aztreonam (ATM-30), Tobramycin (TOB-10), Tazobactam (TZP-110), Ceftriaxone (CRO-30), and Cefuroxime sodium (CXM-30). On the other hand, Pipemidic acid (PIP-20), Gentamicin (CN-10), and Enrofloxacin (ENR-10) showed inhibition of SWAB-1 (11.0 ± 0.0 mm, 10.0 ± 0.0 mm, 14.0 ± 0.0 mm) and SWAB-2 (17.0 ± 0.0 mm, 33.0 ± 0.0 mm, and 28.0 ± 0.0 mm). Similarly, Enrofloxacin (ENR-10) showed maximum inhibition of SWAB-7 (32.0 ± 0.0 mm), SWAB-10 (26.0 ± 0.0 mm), SWAB-11 (25.0 ± 0.0 mm), SWAB-14 (30.0 ± 0.0 mm), and SWAB-15 (29.0 ± 0.0 mm). Gentamicin (CN-10) also showed the inhibition of SWAB-8 (9.0 ± 0.0 mm), SWAB-10 (10.0 ± 0.0 mm), and SWAB-11 (12.0 ± 0.0 mm). Resistogram analysis results revealed that all SWABs were cadmium, chromium, and lead resistant. It was determined that all SWABs were heavy metals and antibiotic-resistant bacteria (Supplementary Figs 1 and 2).

**Table 2:** Antibiogram analysis of spring water-associated bacteria via agar disc diffusion method.

Bacterial IDs↓ Antibiotics used→	Zone of inhibition in mm (M ± SD)									
	(AMC-30)	(ATM-30)	(PIP-20)	(TOB-10)	(CTX-30)	(TZP-110)	(CN-10)	(CRO-30)	(ENR-10)	(CXM-30)
**SWAB-1**	0.0 ± 0.0	0.0 ± 0.0	**11.0 ± 0.0**	0.0 ± 0.0	0.0 ± 0.0	0.0 ± 0.0	**14.0 ± 0.0**	0.0 ± 0.0	**33.0 ± 0.0**	0.0 ± 0.0
**SWAB-2**	0.0 ± 0.0	0.0 ± 0.0	**10.0 ± 0.0**	0.0 ± 0.0	0.0 ± 0.0	0.0 ± 0.0	**17.0 ± 0.0**	0.0 ± 0.0	**28.0 ± 0.0**	0.0 ± 0.0
**SWAB-3**	0.0 ± 0.0	0.0 ± 0.0	0.0 ± 0.0	0.0 ± 0.0	0.0 ± 0.0	0.0 ± 0.0	0.0 ± 0.0	0.0 ± 0.0	0.0 ± 0.0	0.0 ± 0.0
**SWAB-4**	0.0 ± 0.0	0.0 ± 0.0	0.0 ± 0.0	0.0 ± 0.0	0.0 ± 0.0	0.0 ± 0.0	0.0 ± 0.0	0.0 ± 0.0	0.0 ± 0.0	0.0 ± 0.0
**SWAB-5**	0.0 ± 0.0	0.0 ± 0.0	0.0 ± 0.0	0.0 ± 0.0	0.0 ± 0.0	0.0 ± 0.0	0.0 ± 0.0	0.0 ± 0.0	0.0 ± 0.0	0.0 ± 0.0
**SWAB-6**	0.0 ± 0.0	0.0 ± 0.0	0.0 ± 0.0	0.0 ± 0.0	0.0 ± 0.0	0.0 ± 0.0	0.0 ± 0.0	0.0 ± 0.0	0.0 ± 0.0	0.0 ± 0.0
**SWAB-7**	0.0 ± 0.0	0.0 ± 0.0	0.0 ± 0.0	0.0 ± 0.0	0.0 ± 0.0	0.0 ± 0.0	0.0 ± 0.0	0.0 ± 0.0	**32.0 ± 0.0**	0.0 ± 0.0
**SWAB-8**	0.0 ± 0.0	0.0 ± 0.0	0.0 ± 0.0	0.0 ± 0.0	0.0 ± 0.0	0.0 ± 0.0	**9.0 ± 0.0**	0.0 ± 0.0	0.0 ± 0.0	0.0 ± 0.0
**SWAB-9**	0.0 ± 0.0	0.0 ± 0.0	0.0 ± 0.0	0.0 ± 0.0	0.0 ± 0.0	0.0 ± 0.0	0.0 ± 0.0	0.0 ± 0.0	0.0 ± 0.0	0.0 ± 0.0
**SWAB-10**	0.0 ± 0.0	0.0 ± 0.0	**11.0 ± 0.0**	0.0 ± 0.0	0.0 ± 0.0	0.0 ± 0.0	**10.0 ± 0.0**	0.0 ± 0.0	**26.0 ± 0.0**	0.0 ± 0.0
**SWAB-11**	0.0 ± 0.0	0.0 ± 0.0	0.0 ± 0.0	0.0 ± 0.0	**6.0 ± 0.0**	0.0 ± 0.0	**12.0 ± 0.0**	0.0 ± 0.0	**25.0 ± 0.0**	0.0 ± 0.0
**SWAB-12**	0.0 ± 0.0	0.0 ± 0.0	0.0 ± 0.0	0.0 ± 0.0	0.0 ± 0.0	0.0 ± 0.0	0.0 ± 0.0	0.0 ± 0.0	0.0 ± 0.0	0.0 ± 0.0
**SWAB-13**	0.0 ± 0.0	0.0 ± 0.0	0.0 ± 0.0	0.0 ± 0.0	0.0 ± 0.0	0.0 ± 0.0	0.0 ± 0.0	0.0 ± 0.0	0.0 ± 0.0	0.0 ± 0.0
**SWAB-14**	0.0 ± 0.0	0.0 ± 0.0	**5.0 ± 0.0**	0.0 ± 0.0	0.0 ± 0.0	0.0 ± 0.0	0.0 ± 0.0	0.0 ± 0.0	**30.0 ± 0.0**	0.0 ± 0.0
**SWAB-15**	0.0 ± 0.0	0.0 ± 0.0	0.0 ± 0.0	0.0 ± 0.0	0.0 ± 0.0	0.0 ± 0.0	0.0 ± 0.0	0.0 ± 0.0	**29.0 ± 0.0**	0.0 ± 0.0
**SWAB-16**	0.0 ± 0.0	0.0 ± 0.0	0.0 ± 0.0	0.0 ± 0.0	0.0 ± 0.0	0.0 ± 0.0	0.0 ± 0.0	0.0 ± 0.0	0.0 ± 0.0	0.0 ± 0.0
**SWAB-17**	0.0 ± 0.0	0.0 ± 0.0	0.0 ± 0.0	0.0 ± 0.0	0.0 ± 0.0	0.0 ± 0.0	0.0 ± 0.0	0.0 ± 0.0	0.0 ± 0.0	0.0 ± 0.0
**SWAB-18**	0.0 ± 0.0	0.0 ± 0.0	0.0 ± 0.0	0.0 ± 0.0	0.0 ± 0.0	0.0 ± 0.0	0.0 ± 0.0	0.0 ± 0.0	0.0 ± 0.0	0.0 ± 0.0
**SWAB-19**	0.0 ± 0.0	0.0 ± 0.0	0.0 ± 0.0	0.0 ± 0.0	0.0 ± 0.0	0.0 ± 0.0	0.0 ± 0.0	0.0 ± 0.0	0.0 ± 0.0	0.0 ± 0.0

Amoxycillin (AMC-30), Aztreonam (ATM-30), Pipemidic acid (PIP-20), Tobramycin (TOB-10), Cefotaxime (CTX-30), Tazobactam (TZP-110), Gentamicin (CN-10), Ceftriaxone (CRO-30), Enrofloxacin (ENR-10), Cefuroxime sodium (CXM-30)

### 3.4. Molecular Characterization

The range of amplified PCR products was recorded as 932 bps- 1240 bps (Table III). Extracted DNA from some samples, SWAB-16 to SWAB-19, was not of good quality, showed amplification failure, and didn’t proceed further. The initial partial sequences were generated for BLAST analysis at the National Center for Biotechnology Information (NCBI), and the homology percentage was recorded for all spring water-associated bacterial isolates (Table 3).

**Table 3:** BLAST Analysis of spring water-associated bacterial strains.

Bacterial code Id	Scientific Name	Total Score	Query Cover	E value	Per. ident	Acc. Len	Accession
SWAB-1 (1192 bps)	*uncultured Bacillus sp.*	2183	99%	0	99.75	1458	KT600022.1
	*Bacillus subtilis*	2183	99%	0	99.67	1459	AY172513.1
	*Bacillus velezensis*	2183	99%	0	99.83	1453	MT383653.1
	***Bacillus amyloliquefaciens***	2182	99%	0	99.75	1459	MN630201.1
	*Bacillus subtilis*	2182	99%	0	99.75	1439	MN069579.1
	*Bacillus siamensis*	2178	98%	0	99.83	1462	MT672502.1
SWAB-2 (932 bps)	*Bacillus sp. (in: firmicutes)*	1531	70%	0	96.47	1448	MN148884.1
	*Bacillus cereus*	1519	69%	0	96.35	1409	KJ781404.1
	***Bacillus subtilis***	1496	69%	0	96.02	1485	MG914065.1
	*Bacillus cereus*	1500	68%	0	96.41	1413	KU922293.1
	***Bacillus cereus***	1500	69%	0	96.02	1436	KF624695.1
	*Bacillus cereus*	1498	69%	0	96.11	1118	KC441793.1
SWAB-3 (1210 bps)	*Bacillus cereus*	1814	99%	0	94.05	1438	MH633904.1
	***Bacillus subtilis***	1772	98%	0	93.54	1485	JX188065.1
	*Bacillus sp. (in: firmicutes)*	1788	99%	0	93.72	1448	MN148884.1
	*Bacillus cereus*	1786	98%	0	93.78	1429	FJ393296.1
	***Bacillus subtilis***	1772	98%	0	93.54	1485	MG914065.1
	*Bacillus anthracis*	1781	98%	0	93.69	1484	OM236460.1
SWAB-4 (969 bps)	*Bacillus sp. (in: firmicutes)*	1686	87%	0	96.5	1024	OP990756.1
	*Bacillus cereus*	1658	82%	0	97.63	1438	MH633904.1
	*Bacillus cereus*	1654	81%	0	97.72	1419	OL629646.1
	*Bacillus anthracis*	1648	92%	0	95.09	1412	PP129759.1
	*Bacillus sp. (in: firmicutes)*	1634	82%	0	97.22	1448	MN148884.1
	*Bacillus thuringiensis*	1634	92%	0	94.98	1118	MH921613.1
	***Bacillus anthracis***	1634	81%	0	97.41	1484	OM236460.1
	*Bacillus cereus*	1623	79%	0	97.63	1409	KJ781404.1
SWAB-5 (1240 bps)	*Bacillus albus*	1400	99%	0	87.91	1460	OM232457.1
	*Bacillus anthracis*	1310	98%	0	86.05	1439	MN330086.1
	*Bacillus cereus*	1288	99%	0	86.32	1456	MG598445.1
	*Bacillus cereus*	1275	97%	0	86.46	1405	MG516207.1
	*Bacillus cereus*	1262	97%	0	86.25	1412	MK559557.1
	*Bacillus sp. YXA3–25*	1264	99%	0	85.98	1453	JF701942.1
	***Bacillus sp. (in: firmicutes)***	1260	95%	0	87.09	1186	MN190173.1
SWAB-6 (1209 bps)	***Bacillus cereus***	1877	99%	0	95.04	1438	MH633904.1
	*Bacillus cereus*	1873	99%	0	95.1	1419	OL629646.1
	*Bacillus anthracis*	1868	99%	0	94.89	1484	OM236460.1
	*Bacillus sp. (in: firmicutes)*	1844	99%	0	94.55	1448	MN148884.1
	***Bacillus cereus***	1842	97%	0	94.97	1413	KU922293.1
	*Bacillus anthracis*	1829	99%	0	94.31	1488	MH475931.1
	*Bacillus subtilis*	1825	99%	0	94.24	1485	MG914065.1
SWAB-7 (1152 BPS)	Bacillus anthracis	1766	99%	0	93.77	1412	PP129759.1
	Bacillus cereus	1753	96%	0	94.26	1169	MH921617.1
	Bacillus cereus	1748	98%	0	93.84	1228	KU196752.1
	***Bacillus anthracis***	1746	99%	0	93.17	1484	OM236460.1
	Bacillus sp. (in: firmicutes)	1744	99%	0	93.15	1448	MN148884.1
	Bacillus sp. S131a	1740	99%	0	93.23	1297	AB909434.1
	Bacillus wiedmannii	1740	99%	0	93.42	1426	OM510278.1
	Bacillus thuringiensis	1735	93%	0	95.23	1118	MH921613.1
SWAB-8 (1112 bps)	***Lysinibacillus fusiformis***	2091	98%	0.0	99.48%	1444	JQ071512.1
	*Lysinibacillus sphaericus*	2087	98%	0.0	99.39%	1465	MF000302.1
	*Lysinibacillus fusiformis*	2089	98%	0.0	99.39%	1454	PP565089.1
	*Lysinibacillus sphaericus*	2087	98%	0.0	99.48%	1514	KF228925.1
	*Bacillus sp. JG-TB11*	2087	98%	0	99.48	1471	FR849923.1
SWAB-9 (1152 bps)	*Lysinibacillus sphaericus*	2087	98%	0.0	99.39%	1465	MF000302.1
	*Lysinibacillus fusiformis*	2091	98%	0	99.48	1444	JQ071512.1
	*Lysinibacillus fusiformis*	2089	98%	0	99.39	1454	PP565089.1
	*Lysinibacillus fusiformis*	2087	98%	0	99.48	1482	GU125642.1
	***Lysinibacillus sp.***	2087	98%	0	99.48	1493	OR902475.1
	*Lysinibacillus fusiformis*	2087	98%	0	99.48	1444	MN826508.1
	*Lysinibacillus sp. VKK-5OL*	2087	98%	0	99.48	1506	JX871464.1
SWAB-10 (1187 bps)	*Bacillus paranthracis*	1807	99%	0.0	94.53%	1453	OM144955.1
	*Bacillus nitratireducens*	1805	98%	0.0	94.58%	1427	MK418697.1
	*Bacillus toyonensis*	1803	99%	0.0	94.51%	1437	MW742349.1
	*Bacillus anthracis*	1812	98%	0.0	94.97%	1397	KJ535337.1
	*Bacillus paramycoides*	1801	98%	0.0	94.79%	1419	OP853073.1
	*Bacillus clarus*	1797	99%	0.0	94.43%	1451	OR398809.1
	*Bacillus tropicus*	1796	99%	0.0	94.36%	1539	PQ319730.1
	*Bacillus pacificus*	1796	99%	0.0	94.36%	1551	PP907058.1
	***Bacillus wiedmannii***	1740	99%	0	93.42	1426	OM510278.1
	*Bacillus thuringiensis*	1735	93%	0	95.23	1118	MH921613.1
SWAB-11 (1203 bps)	***uncultured Bacillus sp.***	2183	99%	0	99.75	1458	KT600022.1
	*Bacillus amyloliquefaciens*	2182	99%	0	99.75	1459	MN630201.1
	*Bacillus subtilis*	2182	99%	0	99.75	1439	MN069579.1
	*Bacillus velezensis*	2183	99%	0	99.83	1453	MT383653.1
	*Bacillus siamensis*	2178	98%	0	99.83	1462	MT672502.1
	*Bacillus subtilis*	2183	99%	0	99.67	1459	AY172513.1
SWAB-12 1035 bps)	*Bacillus thuringiensis*	1755	99%	0.0	93.16%	1461	MN509082.1
	*Bacillus nitratireducens*	1748	99%	0.0	93.12%	1427	MK418697.1
	*Bacillus cereus*	1746	97%	0.0	93.66%	1411	ON510011.1
	*Bacillus tropicus*	1742	99%	0.0	92.99%	1449	PQ432893.1
	***Bacillus anthracis***	1742	98%	0.0	93.11%	1488	MH475931.1
	*Bacillus paramycoides*	1742	98%	0.0	93.11%	1473	OP024041.1
	*Bacillus pacificus*	1742	98%	0.0	93.11%	1551	PP907058.1
SWAB-13 (1152 bps)	*Bacillus wiedmannii*	1740	99%	0.0	93.42%	1426	OM510278.1
	*Bacillus thuringiensis*	1731	93%	0.0	95.07%	1127	MH921667.1
	*Bacillus cereus*	1753	96%	0.0	94.26%	1169	MH921617.1
	*Bacillus proteolyticus*	1716	96%	0.0	93.68%	1173	MH921595.1
	*Bacillus tropicus*	1703	93%	0.0	94.70%	1125	MH921620.1
	*Bacillus paramycoides*	1701	99%	0.0	92.51%	1448	OR098477.1
	***Bacillus weihenstephanensis***	1698	94%	0.0	94.02%	1170	EU161999.1
	*Bacillus paranthracis*	1696	99%	0.0	92.44%	1453	OM144955.1
	*Bacillus toyonensis*	1692	99%	0.0	92.42%	1437	MW742349.1
SWAB-14 (986 bps)	*Flavobacterium columnare*	1114	91%	0.0	85.18%	1151	KF051089.1
	*Neobacillus novalis*	1109	97%	0.0	83.97%	1406	MG516166.1
	*Bacillus wiedmannii*	1103	99%	0.0	83.63%	1488	OP020876.1
	***Bacillus thuringiensis***	1098	99%	0.0	83.55%	1515	FJ236808.1
	*Bacillus tropicus*	1160	98%	0.0	84.67%	1436	ON844106.1
	*Bacillus albus*	1136	77%	0.0	89.10%	924	PQ097131.1
	*Bacillus paramycoides*	1168	99%	0.0	84.66%	1436	ON693785.1
	*Bacillus anthracis*	1310	98%	0.0	86.05%	1439	MN330086.1
SWAB-15 (1187 bps)	*Bacillus subtilis*	1840	99%	0	95.03	1485	LC469932.1
	*Bacillus cereus*	1832	99%	0	94.93	1438	MH633904.1
	*Bacillus sp. (in: firmicutes)*	1832	99%	0	94.94	1448	MN148884.1
	*Bacillus cereus*	1829	99%	0	94.86	1429	FJ393296.1
	***Bacillus cereus***	1829	98%	0	95	1419	OL629646.1
	*Bacillus subtilis*	1825	99%	0	94.79	1485	MG914065.1
	*Bacillus cereus*	1821	98%	0	94.99	1429	GQ501070.1
	*Bacillus cereus*	1818	99%	0	94.7	1442	OQ940650.1
	*Bacillus thuringiensis*	1816	99%	0	94.69	1468	OP420549.1
SWAB-16	Bad quality DNA						
SWAB-17	Bad quality DNA						
SWAB-18	Bad quality DNA						
SWAB-19	Bad quality DNA						

### 3.5. Phylogenetic analysis

On the other hand, A phylogenetic tree was generated using the 16S rRNA sequences obtained from all spring water-associated bacterial isolates, along with BLAST nucleotide sequences. The results indicated that all bacterial isolates exhibited similarity to BLAST analysis (Supplementary Figs 3-17 in S1 File). The phylogenetic relationships between the bacterial isolates and BLAST sequences were analyzed using the Maximum Likelihood method based on the Tamura-Nei model. Results showed that spring water-associted bacteria were closely related to the *Bacillus amyloliquefaciens* (MN630201.1), *Bacillus subtilis* (MG914065.1) and *Bacillus cereus* (KF624695.1), *Bacillus subtilis* (JX188065.1), *Bacillus anthracis* (OM236460.1), Bacillus sp, (in firmicutes) (MN190173.1), *Bacillus cereus* (MH633904.1), *Bacillus anthracis (*OM236460.1), *Lysinibacillus fusiformis* (JQ071512.1), *Lysinibacillus sp* (OR902475.1), *Bacillus wiedmannii* (OM510278.1), *uncultured Bacillus sp.* (KT600022.1), *Bacillus anthracis* (MH475931.1), *Bacillus weihenstephanensis* (EU161999.1), *Bacillus thuringiensis (*FJ236808.1), and *Bacillus cereus (*FJ393296.1) supporting the 100% value from bootstrap analysis of the phylogenetic trees (Supplementary Figs 3-17 in S1 File).

### 3.6. Bacterial health risk assessment

The ingested dose of Bacillus species in relation to the water consumption by adults and children (2 L/person/day for adults and 1 L/person/day for children) was recorded. The mean ingested concentrations of Bacillus species via drinking water were recorded in the range of 1560 CFU/100 ml to 4380 CFU/100 ml in adults and 780 CFU/100 ml to 2190 CFU/100 ml in children, respectively (Table 4). The risk of infection/day and annual risk of infection for both adults and children due to consumption of contaminated spring water was also estimated. Results revealed that the spring water consumption from CSW-24 spring showed the highest mean risk infection/day values of 0.53% for adults and 0.27% for children through the exponential model. Similarly, the highest mean risk infection/day was also calculated for CSW-22 (0.47 for adults and 0.23 for children), CSW-20 (0.44 for adults and 0.22 for children), CSW-23 (0.44 for adults and 0.22 for children), CSW-21 (0.43 for adults and 0.21 for children), and CSW-1 (0.40 for adults and 0.20 for children), respectively (Table 5). The highest mean risk infection/day through the beta-poisson model was recorded for CSW-24 (0.349, 0.469)> CSW-22 (0.345, 0.463)> CSW-20 (0.342, 0.460)> CSW-21 (0.342, 0.458)> CSW-23 (0.342, 0.460)> CSW-28 (0.339, 0.455) for both adults and children, respectively (Table 5). It was shown that the Beta-Poisson model generally performs better because it accounts for variability in host susceptibility and pathogen infectivity, making it more flexible and better fitting than the exponential model.

**Table 4:** The ingested dose of *Bacillus species* (ingested volume of water × mean concentration of *Bacillus species* in relation to the water consumption by adults (2 L/person/day) and children (1 L/person/day).

Spring water samples IDs↓ Calculations→	Exposure Assessment					
	Adults			Children		
	Concentration (CFU/100 mL)	Volume of drinking water	Dose ingested per day	Concentration (CFU/100 mL)	Volume of drinking water	Dose ingested per day
**CSW-1**	2000	2	4000	2000	1	2000
**CSW-2**	1000	2	2000	1000	1	1000
**CSW-3**	780	2	1560	780	1	780
**CSW-4**	800	2	1600	800	1	800
**CSW-5**	1000	2	2000	1000	1	1000
**CSW-6**	1270	2	2540	1270	1	1270
**CSW-7**	1400	2	2800	1400	1	1400
**CSW-8**	1300	2	2600	1300	1	1300
**CSW-9**	1230	2	2460	1230	1	1230
**CSW-10**	1450	2	2900	1450	1	1450
**CSW-11**	1468	2	2936	1468	1	1468
**CSW-12**	1459	2	2918	1459	1	1459
**CSW-13**	1376	2	2752	1376	1	1376
**CSW-14**	1789	2	3578	1789	1	1789
**CSW-15**	1560	2	3120	1560	1	1560
**CSW-16**	1567	2	3134	1567	1	1567
**CSW-17**	1235	2	2470	1235	1	1235
**CSW-18**	1457	2	2914	1457	1	1457
**CSW-19**	1678	2	3356	1678	1	1678
**CSW-20**	2190	2	4380	2190	1	2190
**CSW-21**	2134	2	4268	2134	1	2134
**CSW-22**	2341	2	4682	2341	1	2341
**CSW-23**	2190	2	4380	2190	1	2190
**CSW-24**	2671	2	5342	2671	1	2671
**CSW-25**	1897	2	3794	1897	1	1897
**CSW-26**	1768	2	3536	1768	1	1768
**CSW-27**	1875	2	3750	1875	1	1875
**CSW-28**	1982	2	3964	1982	1	1982
**CSW-29**	1890	2	3780	1890	1	1890
**CSW-30**	1567	2	3134	1567	1	1567

**Table 5:** Probability of infection per day and per year, and probability of illness for adults and children due to the consumption of water contaminated with pathogenic *Bacillus* species.

Spring water samples IDs↓ Calculations→	Exponential model		Beta-Poisson model							
	Adults (%)	Children (%)	Adults				Children			
			(*P*_*inf*_) per day	(P_inf.annual_) per year	(*P*_*ill*_) per infection	(*P*_*ill*_) %	(*P*_*inf*_) per day	(P_inf.annual_) per year	(*P*_*ill*_) per infection	(*P*_*ill*_) %
**CSW-1**	0.40	0.20	0.019	1.0	0.50	50.0	0.011	1.0	0.69	68.97
**CSW-2**	0.20	0.10	0.010	1.0	0.49	48.7	0.006	0.9	0.62	61.62
**CSW-3**	0.16	0.08	0.008	0.9	0.47	47.0	0.005	0.8	0.57	56.67
**CSW-4**	0.16	0.08	0.008	0.9	0.47	47.2	0.005	0.8	0.57	57.22
**CSW-5**	0.20	0.10	0.010	1.0	0.49	48.7	0.006	0.9	0.62	61.62
**CSW-6**	0.25	0.13	0.012	1.0	0.49	49.5	0.007	0.9	0.65	65.25
**CSW-7**	0.28	0.14	0.014	1.0	0.50	49.7	0.008	0.9	0.66	66.39
**CSW-8**	0.26	0.13	0.013	1.0	0.50	49.5	0.008	0.9	0.66	65.54
**CSW-9**	0.25	0.12	0.012	1.0	0.49	49.4	0.007	0.9	0.65	64.84
**CSW-10**	0.29	0.14	0.014	1.0	0.50	49.7	0.008	1.0	0.67	66.74
**CSW-11**	0.29	0.15	0.014	1.0	0.50	49.7	0.008	1.0	0.67	66.87
**CSW-12**	0.29	0.15	0.014	1.0	0.50	49.7	0.008	1.0	0.67	66.81
**CSW-13**	0.27	0.14	0.013	1.0	0.50	49.6	0.008	0.9	0.66	66.20
**CSW-14**	0.36	0.18	0.017	1.0	0.50	49.9	0.010	1.0	0.68	68.40
**CSW-15**	0.31	0.16	0.015	1.0	0.50	49.8	0.009	1.0	0.67	67.41
**CSW-16**	0.31	0.16	0.015	1.0	0.50	49.8	0.009	1.0	0.67	67.45
**CSW-17**	0.25	0.12	0.012	1.0	0.49	49.4	0.007	0.9	0.65	64.89
**CSW-18**	0.29	0.15	0.014	1.0	0.50	49.7	0.008	1.0	0.67	66.79
**CSW-19**	0.34	0.17	0.334	1.0	0.50	50.0	0.447	1.0	0.70	70.00
**CSW-20**	0.44	0.22	0.342	1.0	0.50	50.0	0.460	1.0	0.70	70.00
**CSW-21**	0.43	0.21	0.342	1.0	0.50	50.0	0.458	1.0	0.70	70.00
**CSW-22**	0.47	0.23	0.345	1.0	0.50	50.0	0.463	1.0	0.70	70.00
**CSW-23**	0.44	0.22	0.342	1.0	0.50	50.0	0.460	1.0	0.70	70.00
**CSW-24**	0.53	0.27	0.349	1.0	0.50	50.0	0.469	1.0	0.70	70.00
**CSW-25**	0.38	0.19	0.338	1.0	0.50	50.0	0.453	1.0	0.70	70.00
**CSW-26**	0.35	0.18	0.335	1.0	0.50	50.0	0.450	1.0	0.70	70.00
**CSW-27**	0.37	0.19	0.337	1.0	0.50	50.0	0.453	1.0	0.70	70.00
**CSW-28**	0.40	0.20	0.339	1.0	0.50	50.0	0.455	1.0	0.70	70.00
**CSW-29**	0.38	0.19	0.338	1.0	0.50	50.0	0.453	1.0	0.70	70.00
**CSW-30**	0.31	0.16	0.331	1.0	0.50	50.0	0.444	1.0	0.70	70.00

The mean risk of infection/year due to the presence of *Bacillus species* ranged between 0.9 to 1.0 for the annual risk of infection for both adults and children. These values exceeded the acceptable risk value (10^−4^) for all the studied spring water and indicating that health risks are probably occurring from the exposure to *Bacillus species* by consumption of spring water. Similarly, the highest mean risk of illness (%) due to Bacillus species was recorded in the range of 56.67% to 70.0% in children compared to the adults, as 47.0% to 50.0% (Table 5).

#### *In vitro* antibacterial effect of phytobiosorbents.

The antibacterial effect of seeds, roots, and aerial parts of *B. rapa* and *S. oleracea* was screened against all identified SWABs through the agar well diffusion method, and results revealed that aerial parts of *S. oleracea* showed the susceptible and intermediate inhibition of bacteria in the range of 4.0 ± 0.0 mm to 10.0 ± 0.0 mm. The significant (p ≤ 0.05, p ≤ 0.01, p ≤ 0.001) difference among all treatments were recorded. On the other hand, *Spinacia oleracea* root aqueous extract showed significant (p ≤ 0.001) and maximum inhibition of all identified *Bacillus* species compared to seed extracts except SWAB-13, SWAB-14, SWAB-15, and SWAB-16. The significant (p ≤ 0.001) and maximum zone of inhibition of SWAB-7 (15.0 ± 0.0 mm) and SWAB-19 (15.0 ± 0.0 mm) was noted, while the minimum inhibition of SWAB-13 (9.0 ± 0.0 mm) was recorded, respectively (Table 6). Similarly, all SWABs showed resistance against aerial parts of *B. rapa,* while the root extract of *B. rapa* showed the significant (≤0.01, p ≤ 0.001) and maximum inhibition of all tested spring water-associated bacteria in the range of 10.0 ± 0.0 mm to 19.0 ± 0.0 mm. The zone of inhibition of SWAB-3 (11.0 ± 0.0 mm), SWAB-4 (10.0 ± 0.0 mm), SWAB-5 (10.0 ± 0.0 mm), SWAB-7 (11.0 ± 0.0 mm), SWAB-8 (12.0 ± 0.0 mm), SWAB-9 (11.0 ± 0.0 mm), SWAB-10 (10.0 ± 0.0 mm), SWAB-11 (11.0 ± 0.0 mm), SWAB-12 (11.0 ± 0.0 mm), SWAB-13 (12.0 ± 0.0 mm), SWAB-14 (10.0 ± 0.0 mm), SWAB-15 (11.0 ± 0.0 mm), SWAB-16 (11.0 ± 0.0 mm), and SWAB-17 (14.0 ± 0.0 mm) was recorded when seeds extract of *B. rapa* was applied (Supplementary Fig 18). Findings illustrated that *B. rapa* and *S. oleracea* could be used as biosorbents to decontaminate the contaminated spring water (Table 6).

**Table 6:** Antibacterial effect of *Brassica rapa* and *Spinach oleracea* against spring water-associated bacteria.

Bacterial IDs↓ Extracts of biosorbents→	Zone of inhibition in mm (M ± SD)					
	*Spinach oleracea*			*Brassica rapa*		
	Seeds	Roots	Aerial parts	Seeds	Roots	Aerial parts
SWAB-1	10.0 ± 0.0	12.0 ± 0.0^aa,bbb,ccc^	6.0 ± 0.0^aaa,bbb^	7.0 ± 0.0	17.0 ± 0.0^aaa,bbb,ccc^	3.0 ± 0.0^aaa,bbb^
SWAB-2	10.0 ± 0.0	13.0 ± 0.0^aa,bbb,ccc^	7.0 ± 0.0^aaa,bbb^	8.0 ± 0.0	17.0 ± 0.0^aaa,bbb,ccc^	4.0 ± 0.0^aaa,bbb^
SWAB-3	10.0 ± 0.0	12.0 ± 0.0^aa,bbb,ccc^	5.0 ± 0.0^aa,bb^	11.0 ± 0.0	15.0 ± 0.0^aaa,bbb,ccc^	6.0 ± 0.0^aaa,bbb^
SWAB-4	10.0 ± 0.0	13.0 ± 0.0^aa,bb,cc^	10.0 ± 0.0^bbb^	10.0 ± 0.0	15.0 ± 0.0^aaa,bbb,ccc^	6.0 ± 0.0^aaa,bbb^
SWAB-5	9.0 ± 0.0	11.0 ± 0.0^aa,bb,cc^	9.0 ± 0.0^bbb^	10.0 ± 0.0	16.0 ± 0.0^aaa,bbb,ccc^	4.0 ± 0.0^aaa,bbb^
SWAB-6	13.0 ± 0.0	14.0 ± 0.0^aa,bbb,ccc^	8.0 ± 0.0 ^aaa,bbb^	8.0 ± 0.0	10.0 ± 0.0^aa,bb,ccc^	3.0 ± 0.0^aaa,bbb^
SWAB-7	11.0 ± 0.0	15.0 ± 0.0^aa,bbb,ccc^	5.0 ± 0.0^aaa,bbb^	11.0 ± 0.0	19.0 ± 0.0^aaa,bbb,ccc^	0.0 ± 0.0
SWAB-8	9.0 ± 0.0	12.0 ± 0.0^aa,bbb,ccc^	7.0 ± 0.0^a,bbb^	12.0 ± 0.0	14.0 ± 0.0^aa,bb,ccc^	0.0 ± 0.0
SWAB-9	10.0 ± 0.0	11.0 ± 0.0^aa,bb,ccc^	9.0 ± 0.0^a,bbb^	11.0 ± 0.0	13.0 ± 0.0 ^aa,bb,ccc^	0.0 ± 0.0
SWAB-10	7.0 ± 0.0	12.0 ± 0.0^aa,bb,cc^	9.0 ± 0.0 ^a,bbb^	10.0 ± 0.0	17.0 ± 0.0^aaa,bbb,ccc^	0.0 ± 0.0
SWAB-11	9.0 ± 0.0	11.0 ± 0.0^aa,bbb,ccc^	4.0 ± 0.0 ^aaa,bbb^	11.0 ± 0.0	19.0 ± 0.0^aaa,bbb,ccc^	0.0 ± 0.0
SWAB-12	6.0 ± 0.0	14.0 ± 0.0^aaa,bbb,ccc^	6.0 ± 0.0^bbb^	11.0 ± 0.0	19.0 ± 0.0^aaa,bbb,ccc^	3.0 ± 0.0^aaa,bbb^
SWAB-13	6.0 ± 0.0	9.0 ± 0.0^aa,bb,cc^	6.0 ± 0.0^bbb^	12.0 ± 0.0	15.0 ± 0.0^aaa,bbb,ccc^	0.0 ± 0.0
SWAB-14	10.0 ± 0.0	10.0 ± 0.0^b,c^	9.0 ± 0.0^b^	10.0 ± 0.0	14.0 ± 0.0 ^aa,bb,ccc^	0.0 ± 0.0
SWAB-15	11.0 ± 0.0	11.0 ± 0.0^c^	10.0 ± 0.0^b^	11.0 ± 0.0	18.0 ± 0.0^aaa,bbb,ccc^	0.0 ± 0.0
SWAB-16	11.0 ± 0.0	11.0 ± 0.0^c^	10.0 ± 0.0^b^	11.0 ± 0.0	16.0 ± 0.0^aaa,bbb^	2.0 ± 0.0^aaa,bbb^
SWAB-17	8.0 ± 0.0	12.0 ± 0.0^aa,bbb,ccc^	7.0 ± 0.0^bbb^	14.0 ± 0.0	15.0 ± 0.0 ^a,bb,ccc^	3.0 ± 0.0^aaa,bbb^
SWAB-18	8.0 ± 0.0	13.0 ± 0.0^aa,bbb,ccc^	5.0 ± 0.0^aa,bbb^	9.0 ± 0.0	18.0 ± 0.0^aaa,bbb^	5.0 ± 0.0^aaa,bbb^
SWAB-19	10.0 ± 0.0	15.0 ± 0.0^aa,bbb^	7.0 ± 0.0^aa,bbb^	9.0 ± 0.0	16.0 ± 0.0^aaa,bbb^	0.0 ± 0.0

### 3.7. Ex-situ remediation of spring water

**Fig 1:**
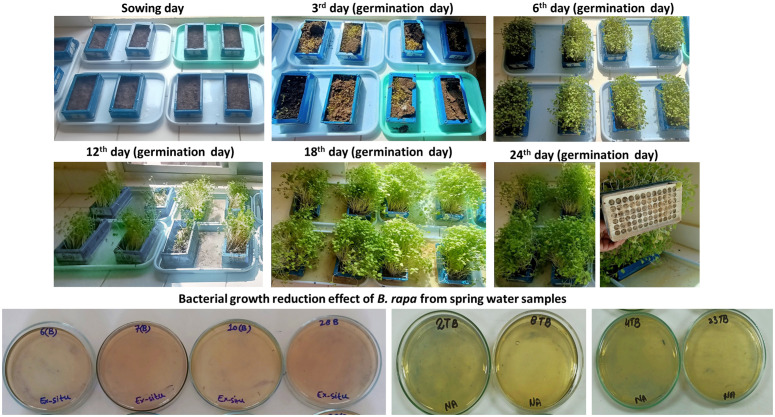
Ex situ bacterial decontamination of spring water using *B. rapa* via wetland technology.

**Fig 2:**
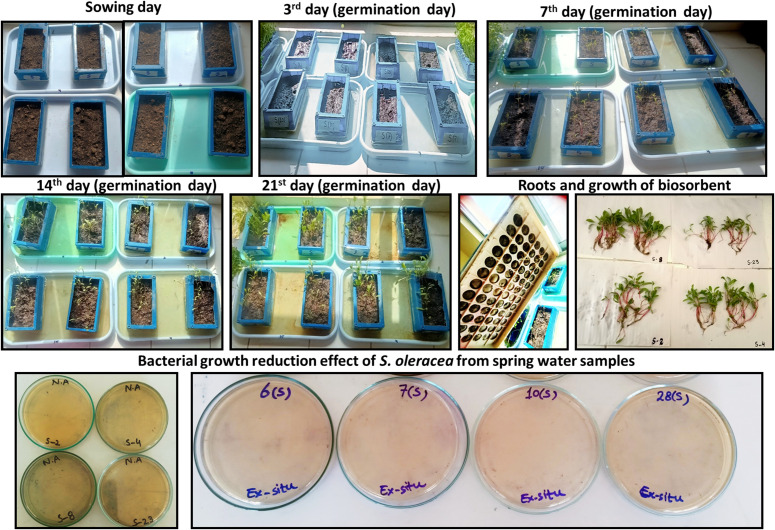
Ex situ bacterial decontamination of spring water using *S. oleracea* via wetland technology.

**Fig 3:**
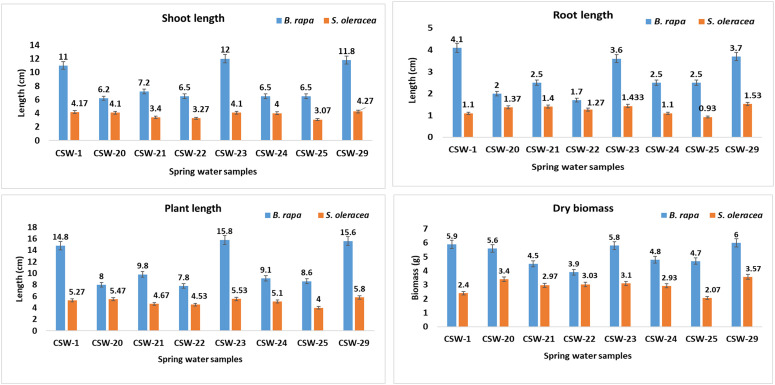
Impact of contaminated spring water on the sprouting and growth of *B. rapa* and *S. oleracea.*

**Fig 4:**
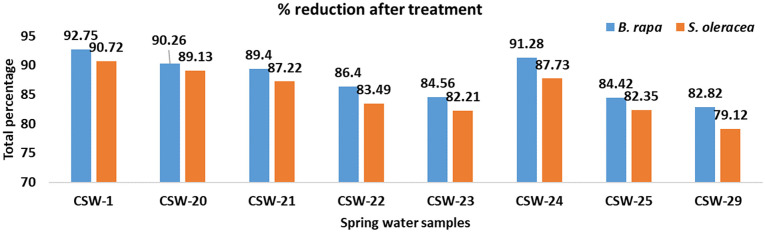
Bacterial growth reduction (%) in spring water samples after *B. rapa* and *S. oleracea* treatments.

The impact of contaminated spring water on the sprouting and growth of *B. rapa* and *S. oleracea* was also evaluated, and results revealed that the tested spring water did not affect the sprouting and growth of *B. rapa* compared to the *S. oleracea* (Figs 1 and 2). Fig 3 also reveals the maximum whole plant length, shoot length, root length, and dry biomass of *B. rapa* compared to *S. oleracea*. *B. rapa* showed maximum seed germination and growth in CSW-2, CSW-8, and CSW-28, respectively. On the other hand, contaminated spring water had an excessive effect on the growth and germination of *S. oleracea*. The number of plants and biomass of *S. oleracea* declined in all treated spring water samples, and the maximum biomass of *B. rapa* was recorded compared to that of *S. oleracea* (Figs 1-3). Following ex-situ remediation experiments, intriguing findings regarding the bactericidal properties of *B. rapa* and *S. oleracea* were also observed (Figs 1 and 2). After treatment significant (p < 0.001) reduction in bacterial growth was noted (Fig 4). *B. rapa* showed maximum reduction of bacterial growth in treated spring water samples such as 92.75% in CSW1, 91.28% in CSW-24, and 90.26% in CSW-20 compared to *S. oleracea* like 90.72% in CSW1, 87.73% in CSW-24, and 89.13% in CSW-20, respectively. After treatment, physicochemical parameters were recorded from all spring water treated samples to check the water quality. The results revealed that all the values were under the permissible values as recommended by Pakistan Standards & Quality Control Authority (PSQCA). The pH (7.67 ± 0.58 to 8.0 ± 0.00) was recorded under recommended permissible values (6.5 to 8.5), temperature (12.4 ± 0.36°C to 13.2 ± 0.26°C), electrical conductivity (245.0 ± 7.54 µS/cm to 365.13 ± 13.89 µS/cm) under recommended permissible values (750 µS/cm), turbidity (0.6 ± 1.0 NTU to 2.6 ± 2.51 NTU) under recommended permissible values of WHO and PSQCA (<5), total dissolved solids (122.0 ± 1.00 ppm to 247.6 ± 3.21 ppm) under recommended permissible values (1000 ppm) dissolved oxygen (4.53 ± 0.42 mg/L to 6.8 ± 0.7 mg/L) under recommended permissible values (10 mg/L), and COD (7.8 ± 0.4 mg/L to 9.8 ± 0.4 mg/L) under recommended permissible values (<10 mg/L) were recorded. On the other hand, BOD (5.1 ± 0.1 mg/L to 5.13 ± 0.15 mg/L) is not under the recommended permissible values of PSQCA (<10 mg/L). Similarly, pH, dissolved oxygen, and BOD were not recorded according to the recommended values by WHO.

## 4. Discussion

### 4.1. Bacterial contamination

In rural regions of the majority of developing nations, where communal water sources are used, bacterial contamination of drinking water is a significant cause of water-borne illnesses [1,9]. The current finding agreed with Meradji et al. [7] and Sarker et al. [63] that water is the source of bacterial contamination. Similarly in the current study, all collected spring water (CSW-1 to CSW-30) from District Muzaffarabad were contaminated with pathogenic bacteria such as *Bacillus amyloliquefaciens*, *Bacillus subtilis, Bacillus cereus*, *Bacillus subtilis*, *Bacillus* *anthracis*, *Bacillus sp, (in firmicutes)*, *Bacillus cereus*, *Bacillus anthracis, Lysinibacillus fusiformis*, *Lysinibacillus sp*, *Bacillus wiedmannii*, *uncultured Bacillus sp., Bacillus anthracis*, *Bacillus weihenstephanensis*, *Bacillus* thuringiensis and *Bacillus cereus*, and they showed the exceeded acceptable risk infection value as recommended by WHO (10^−4^). Our results also concur with the findings reported in Northern Pakistan, Ethiopia, and Zimbabwe, which pointed to highly contaminated drinking water sources with *E. coli* due to poor hygiene practices and neighborhood sanitation [64,65]. Current findings indicate the significant contamination of spring water with *Bacillus spp*., due to anthropogenic activities and improper water usage by the public in sampling areas. The study area is mostly a grazing area, some belongs to small farms, which leads to microbial contamination. Our findings are consistent with [66,67], who also reported the spring water contamination due to wildlife and poor livestock management. The physicochemical parameters of soil, such as soil permeability, humidity, temperature, and fecal contamination, also affect the spring water quality [68].

Previous study revealed that *B. anthracis, B. cereus,* and *B. thuringiensis* can cause food poisoning and numerous illnesses such as abscesses, bacteremia/septicemia, wound and burn infections, ear infections, endocarditis, meningitis, ophthalmitis, osteomyelitis, peritonitis, and respiratory and urinary tract infections [69–71]. Multiple *Bacillus* spp. Pathogenic bacteria cause occasional infections. Anthrax, an acute infection that affects humans, economically significant livestock, and wild animals, is caused by the bacteria *B. anthracis* [71]. Humans can die from anthrax, which can also cause serious lung and gastrointestinal illnesses. One of the food poisoning agents, *B. cereus*, has been documented to infect humans with localized eye and wound infections. Certain strains of *B. thuringiensis* are entomopathogens that have been created as biopesticides and may be able to infect immunocompromised persons [69]. The current finding agrees with the outcomes of Baindara and Aslam [69] that *Bacillus spp.* were α and β hemolytic in nature, and we can say that these are pathogenic bacteria. The pathogenicity of all isolated spring water-associated bacteria (SWAB1-SWAB-19) might be due to the production of numerous enzymes and aggressins.

### 4.2. Antibiotic and heavy metals resistance

In the current research, it was observed that isolated spring water-associated bacteria (SWAB1-SWAB-19) showed resistance against standard antibiotics such as Aztreonam (ATM-30), Tobramycin (TOB-10), Amoxycillin (AMC-30) Piperacillin/Tazobactam (TZP-110), Ceftriaxone (CRO-30), and Cefotaxime (CTX-30), and most of microbes were resistant to Pipemidic acid (PIP-20) and Gentamicin (CN-10) and could be considered as multidrug resistant bacteria, which now recognized as an alarming for the global health. According to the previous literature, urban wastewater discharge, antibiotic and organic waste discharge into the receiving environment can spread antibiotic-resistant bacteria (ARB) and antibiotic-resistant genes (ARGs) into our soils, sediments, and water bodies [72,73]. It was observed that bacteria can develop resistance against antimicrobials (antibiotics), heavy metals, and any biocidal compounds (Disinfectants) when frequently released into the environment [74,75].

In the current study, it was recorded that all SWABs were heavy metal-resistant. We can say that resistant towards antimicrobials/antibiotics and heavy metals may be occurred through several mechanisms such as 1, antibiotic or heavy metals sequestration can block the compounds to reach the target; 2, modification in bacterial membrane structure to protect the bacterium from chemicals, 3. by enhancing resistance gene dete74rminants through co-selection mechanisms, contaminants including biocides and heavy metals can also aid in the spread of AMR [73]. For dangerous substances such as solvents [76], biocides [75,77], heavy metals [75,77], and antibiotics, co-selection of resistance genes has been documented. Co-resistance, in which the selection of one gene promotes the selection of another that typically does not provide a selective advantage to the compound of interest [78], and cross-resistance, in which one resistance gene protects a variety of harmful chemicals [75], are two ways that co-selection can take place. We can say that antibiotic use in livestock and heavy metals present in agricultural runoff create a shared environmental pressure that selects for microbes carrying genes that provide survival advantages. Many bacteria possess co-resistance or cross-resistance mechanisms, where genes for antibiotic resistance and heavy-metal resistance are located together on the same plasmid, transposon, or integron. When heavy metals such as copper, zinc, cadmium, or arsenic accumulate in soil and water through fertilizers, pesticides, and manure runoff, they exert continuous selective pressure. Even in the absence of antibiotics, bacteria exposed to heavy metals must survive, and those carrying metal-resistance genes often linked with antibiotic-resistance genes are favored. Similarly, livestock treated with antibiotics release resistant bacteria and resistance genes into the environment via manure. When these bacteria enter fields or water bodies, they mix with environmental microbes, enabling horizontal gene transfer.

As a result, environments contaminated with agricultural runoff provide a “hotspot” where bacteria exposed to heavy metals indirectly maintain and spread antibiotic-resistance traits. This means heavy-metal pollution can sustain and amplify antibiotic resistance, while antibiotic use in livestock further accelerates the enrichment of multi-resistant bacterial populations. Together, these two pressures create a cycle that increases the prevalence of microorganisms’ resistant to both antibiotics and heavy metals in agricultural ecosystems and downstream water sources.

### 4.3. Phytoremediation and Biocontrol

So, the development of MDRBs is an emerging problem for public health. Therefore, it is important to develop new compounds or therapies to overcome these problems. Various plants have been used against bacterial pathogens due to the presence of antibacterial compounds [79–81]. In the current study, *Brassica rapa* L. (turnip; family *Cruciferae*) and *Spinacia oleracea* L. (*Amaranthaceae*) were used against spring water-associated pathogenic bacteria based on the pharmacological properties such as cholecystitis, diabetes, jaundice, hepatitis, sore throats, and constipation. Similarly, water spinach possesses antidiabetic, anti-inflammatory, diuretic, anticancer [82], antiseptic activities, and antimicrobial properties. Thus, the antibacterial effect was analyzed through the agar well diffusion method, and results revealed that both *B. rapa* and *S. oleraceae* extracts exhibited significant (p < 0.001) antibacterial activity against all SWABs. Current findings agreed with the previous literature, who demonstrated that these plants possessed a variety of secondary metabolites (phenolic compounds like glucosinolates) and showed diverse bioactivities, including hepatoprotective, antioxidant, hypolipidemic, antimicrobial, anticancer, nephroprotective, antidiabetic, analgesic, cardioprotective, and anti-inflammatory effects, *aeruginosa, Staphylococcus aureus, Escherichia coli,* and *Klebsiella pneumoniae* [83–85]. The findings of Olasupo et al. [86] also confirmed the antibacterial efficacy of *S. oleraceae* ethyl acetate crude extract.

Previous studies have demonstrated the antimicrobial potential of *B. rapa* seeds against *Salmonella paratyphi, Pseudomonas aeruginosa, Staphylococcus aureus, Escherichia coli,* and *Klebsiella pneumoniae* [85–88]. Additionally, other Brassica species, such as radish root, kale leaves, and mustard seeds, have exhibited antimicrobial activity against *Bacillus subtilis, Staphylococcus aureus, Salmonella typhimurium, Enterobacter faecalis, Moraxella catarrhalis, Listeria monocytogenes,* and *Escherichia coli* [89–91]. In another research, Akter et al. [92] revealed that brassica-derived AgNPs showed impressive antibacterial activity against *E. coli* and *Enterobacter sp.,* with inhibition zones of 11.1 ± 0.5 mm and 15 ± 0.5 mm, respectively, surpassing some existing green-synthesized AgNPs, and holding promise for consumer product applications.

After *in vitro* antibacterial analysis of both *B. rapa* and *S. oleraceae* against pathogenic bacteria, an *ex-situ* remediation experiment was conducted, and results revealed that both *B. rapa* and *S. oleraceae* showed significant remediation of microbes from polluted spring water. According to Li et al. [93] and Pavithra and Jaikumar [94], demonstrated that plant-based green technology and adsorption treatments are common treatments for the removal of contaminants. The current study reveals that rhizofiltration and rhizodegradation play an important role in the treatment of contaminated spring water. Phytoremediation is relevant to the cleanup of expansive areas where other traditional methods are incredibly ineffective and expensive. The current study reveals that remediation might be due to the production of root exudates as well as to the long fibrous roots. Our findings agreed with Sabreena et al. [95].

## 5. Conclusions, limitations, and future prospects

The current study confirmed the presence of pathogenic *Bacillus* spp. in all analyzed spring water samples. Preventive measures were carried out to reduce microbial contamination using *B. rapa* and *S. oleracea*. *Ex vivo* remediation of contaminated spring water using *B. rapa* and *S. oleracea* is a promising and environmentally friendly technology to remediate the contaminants and could be used as an efficient biosorbent and antibacterial agent. *B. rapa* roots showed maximum bacterial inhibition in the range of 10.0 ± 0.0 mm to 19.0 ± 0.0 mm.

Despite confirming the presence of pathogenic Bacillus spp. in all analyzed spring water samples and demonstrating the promising ex vivo remediation potential of *Brassica rapa* and *Spinacia oleracea*, the present study has several limitations. First, the remediation experiments were conducted under controlled laboratory conditions, which may not fully represent the complex physicochemical and microbial dynamics of natural spring-water systems. Second, the study focused primarily on Bacillus spp., and therefore does not account for the presence or interaction of other pathogenic or opportunistic microorganisms that may coexist in spring water. Third, the biosorption and antibacterial efficiency of *B. rapa* and *S. oleracea* were evaluated ex vivo, limiting conclusions about their long-term effectiveness, regeneration capacity, and stability under continuous flow or in situ conditions. Additionally, variations in plant biomass age, composition, and environmental stress responses were not examined, which could influence remediation performance. Finally, potential release of plant-derived organic compounds and their ecological or health implications were not assessed, highlighting the need for further in vivo, field-scale, and risk-based investigations before large-scale application.

Future research should involve chromatographic profiling of bioactive compounds and metagenomic analysis of resistant genes. Strengthening collaboration between researchers, public health agencies, and local communities will be essential to translate laboratory findings into practical, sustainable solutions for improving drinking water quality and reducing waterborne disease risks.

## Supporting information

S1 File Supplementary Tables and Figures. (DOC)
