# Supplementary material for: Bacterial diversity, quantitative risk assessment of spring waterborne pathogens and their biocontrol through phyto-biosorbents
Source: PLoS One. 2026 Jul 13;21(7):e0349096. doi: 10.1371/journal.pone.0349096 (PMC13362109; doi:10.1371/journal.pone.0349096)
Supplement: S1 File — (DOC) [file pone.0349096.s001.doc]

**Suplementary Table I. Microscopic characteristics of spring water-associated bacteria on different culture media.**

| **Bacterial IDs↓**  **Characteristics→** | **Staining** | | | **Colony morphology** | | | **Cultural media used** | | | | |
| --- | --- | --- | --- | --- | --- | --- | --- | --- | --- | --- | --- |
|  |  |  |  | **Nutrient agar medium** | | | **Blood agar** | **Macconkey agar** | **MSA** | **TCBS** | **EMB** |
|  | **GS** | **ES** | **Shape** | **Shape** | **Type** | **Size** |  |  |  |  |  |
| **SWAB-1** | + | + | Rod | Conv. | Rou. | Med. | α-hemolytic | Yellow colonies | No growth | Mucoid green | Shiny Pinkish |
| **SWAB-2** | + | + | Rod | Conv. | Rou. | Med. | α-hemolytic | Yellow colonies | No growth | Mucoid green | Shiny Pinkish |
| **SWAB-3** | + | + | Rod | Conv. | Rou. | Med. | α-hemolytic | Pink colonies | No growth | Mucoid green | Shiny Pinkish |
| **SWAB-4** | + | + | Rod | Conv. | Rou. | Med. | α-hemolytic | Pink colonies | No growth | No growth | Shiny Pinkish |
| **SWAB-5** | + | + | Rod | Conv. | Rou. | Med. | α-hemolytic | Yellow colonies | No growth | Mucoid green | Shiny Pinkish |
| **SWAB-6** | + | + | Rod | Conv. | Rou. | Med. | α-hemolytic | Pink colonies | No growth | Mucoid green | Shiny Pinkish |
| **SWAB-7** | + | + | Rod | Conv. | Rou. | Med. | β- hemolytic | Yellow colonies | No growth | Mucoid green | Shiny Pinkish |
| **SWAB-8** | + | + | Rod | Conv. | Rou. | Med. | α-hemolytic | Yellow colonies | Yellow colonies | No growth | Shiny Pinkish |
| **SWAB-9** | + | + | Rod | Conv. | Rou. | Med. | β- hemolytic | Pink colonies | Yellow colonies | No growth | Shiny Pinkish |
| **SWAB-10** | + | + | Rod | Conv. | Rou. | Med. | α-hemolytic | Pink colonies | No growth | Yellow colonies | Shiny Pinkish |
| **SWAB-11** | + | + | Rod | Conv. | Rou. | Med. | β- hemolytic | Pink colonies | No growth | Mucoid green | Shiny Pinkish |
| **SWAB-12** | + | + | Rod | Conv. | Rou. | Med. | β- hemolytic | Pink colonies | No growth | No growth | Shiny Pinkish |
| **SWAB-13** | + | + | Rod | Conv. | Rou. | Med. | β- hemolytic | Pink colonies | No growth | Yellow colonies | Shiny Pinkish |
| **SWAB-14** | + | + | Rod | Conv. | Rou. | Med. | α-hemolytic | Pink colonies | No growth | Yellow colonies | Shiny Pinkish |
| **SWAB-15** | + | + | Rod | Conv. | Rou. | Med. | α-hemolytic | Pink colonies | No growth | Mucoid green | Shiny Pinkish |
| **SWAB-16** | + | + | Rod | Conv. | Rou. | Med. | β- hemolytic | Pink colonies | No growth | Yellow colonies | Shiny Pinkish |
| **SWAB-17** | + | + | Rod | Conv. | Rou. | Med. | β- hemolytic | Pink colonies | No growth | Yellow colonies | Shiny Pinkish |
| **SWAB-18** | + | + | Rod | Conv. | Rou. | Med. | β- hemolytic | Pink colonies | No growth | Yellow colonies | Shiny Pinkish |
| **SWAB-19** | + | + | Rod | Conv. | Rou. | Med. | β- hemolytic | Pink colonies | No growth | Yellow colonies | Shiny Pinkish |

Spring water bacteria (SWAB), Gram staining (GS), Endospore staining (ES), Convex (Conv), Medium (Med), Round (Rou) Mannitol salt agar (MSA), Thiosulfate Citrate Bile Salts Sucrose (TCBS), Eosine methylene blue (EMB), Positive (+)

**
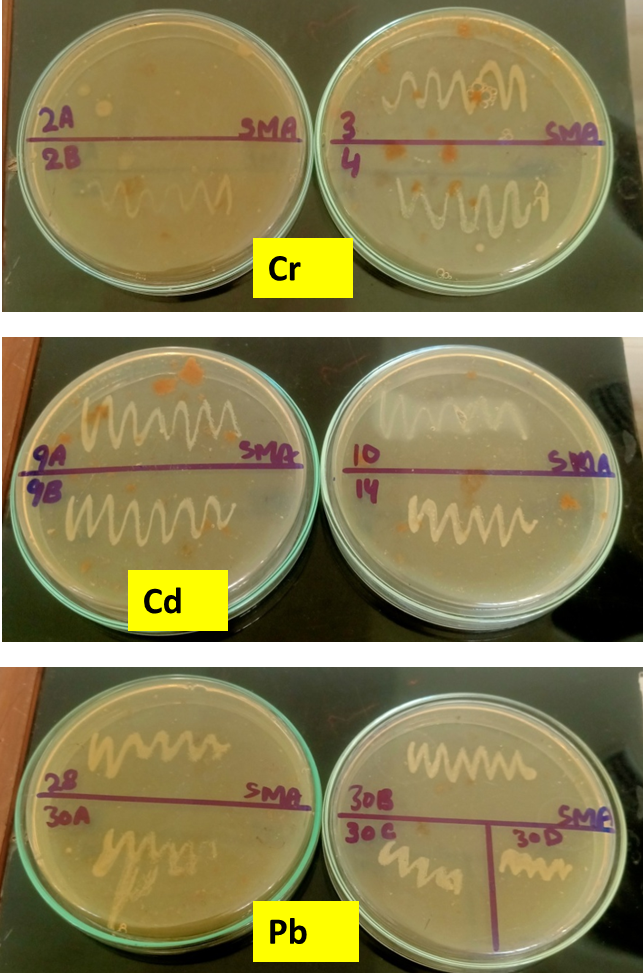
**

**Supplementary Fig 1. Resistogram analysis of tested spring water associated bacteria against cadmium, lead, and chromium containing media.**

**
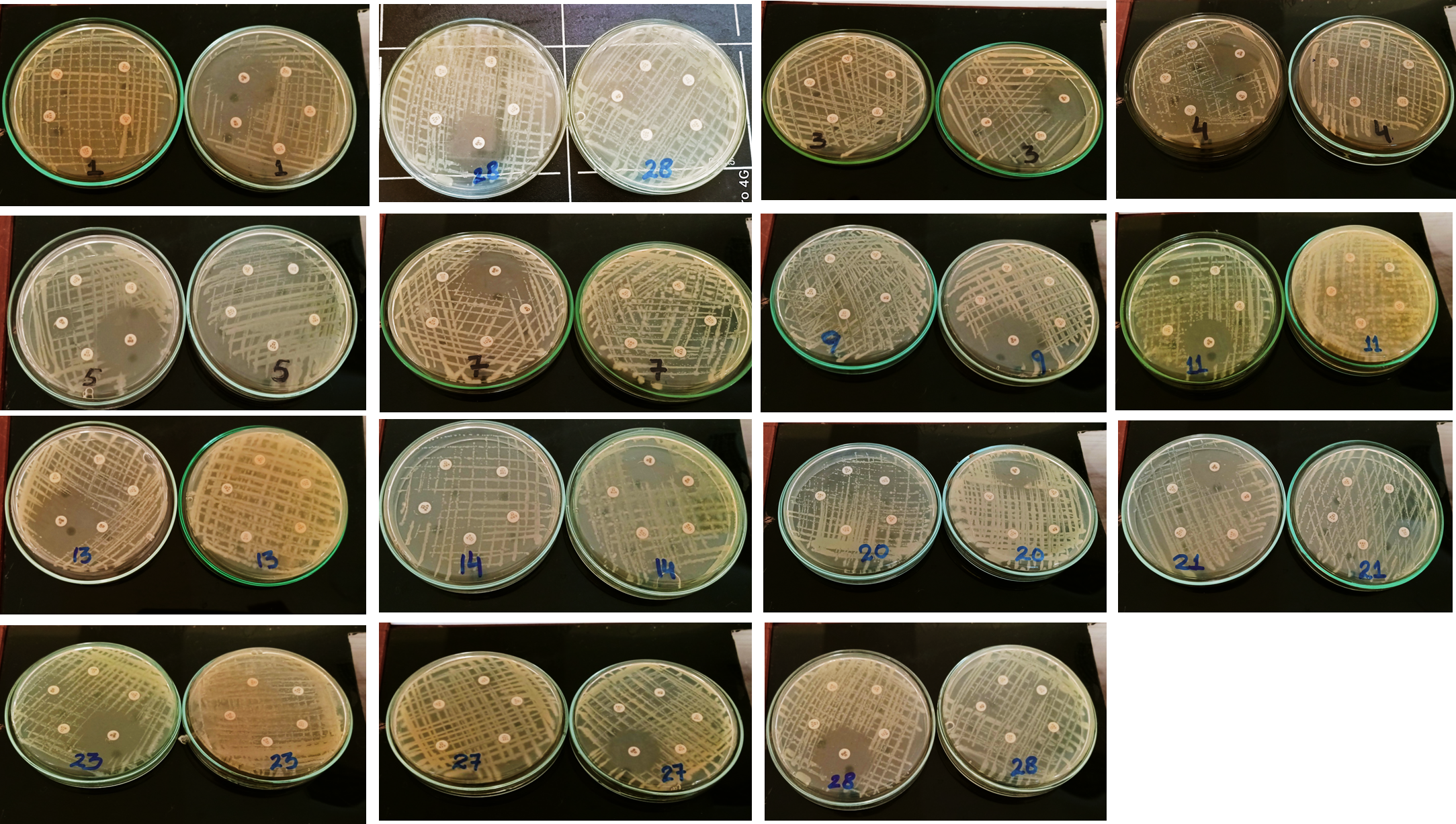
**

**Supplementary Fig 2. Sensitivity and resistance of spring water associated bacteria against standard antibiotics.**

**
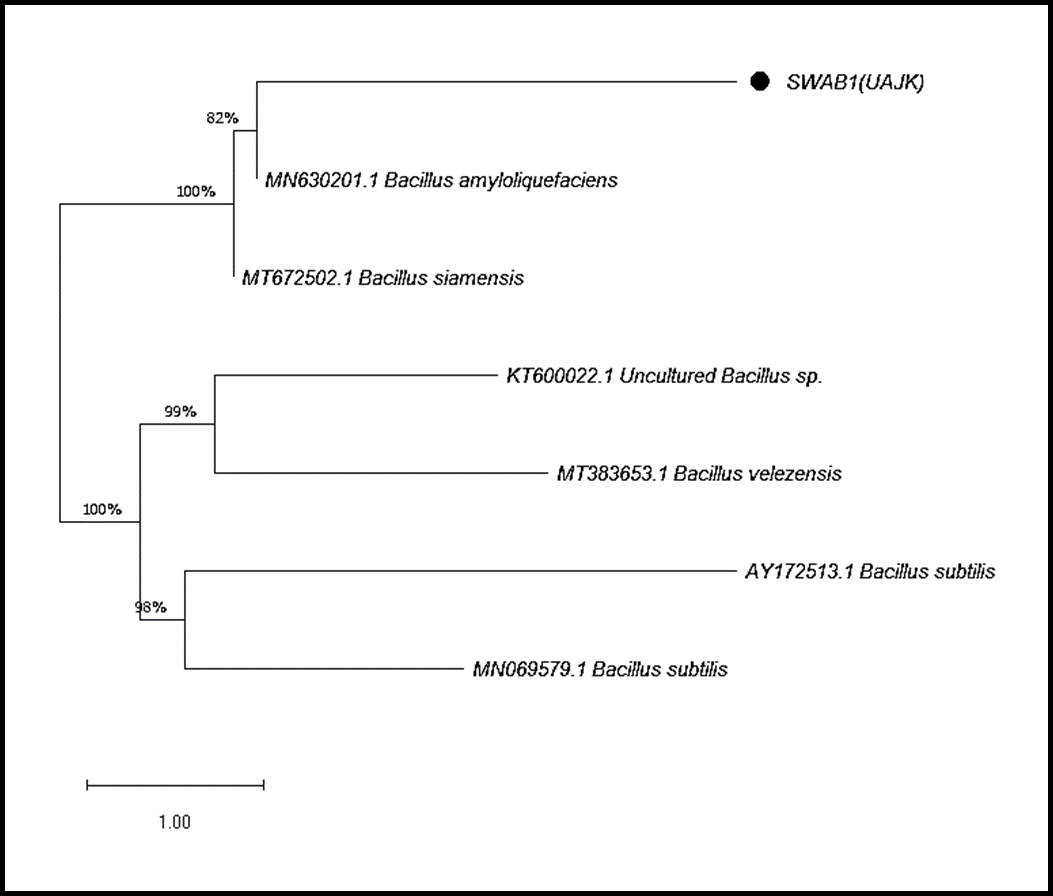
**

**Supplementary Fig 3. Phylogenetic relationship of spring water-associated bacteria SWAB 1 with other NCBI bacterial species.**

**
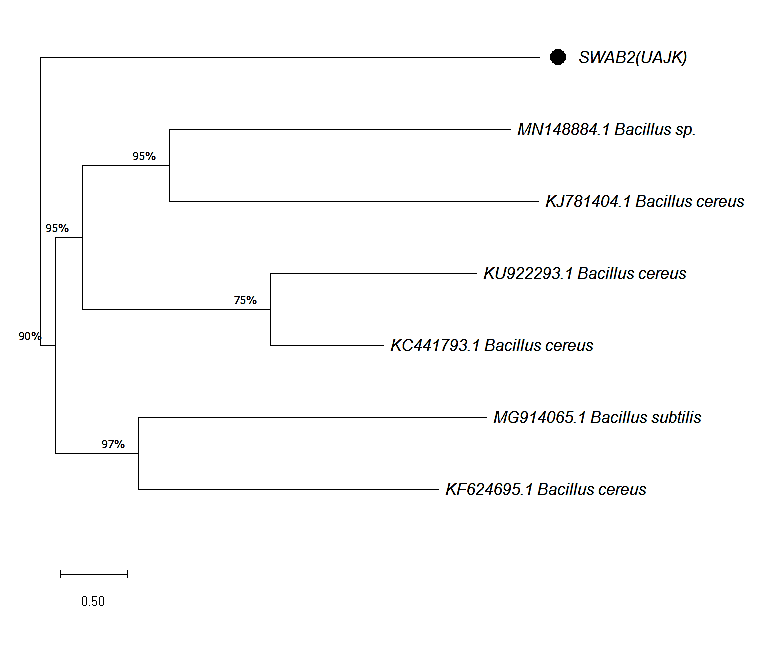
**

**Supplementary Fig 4. Phylogenetic relationship of spring water-associated bacteria SWAB 2 with other NCBI bacterial species.**

**
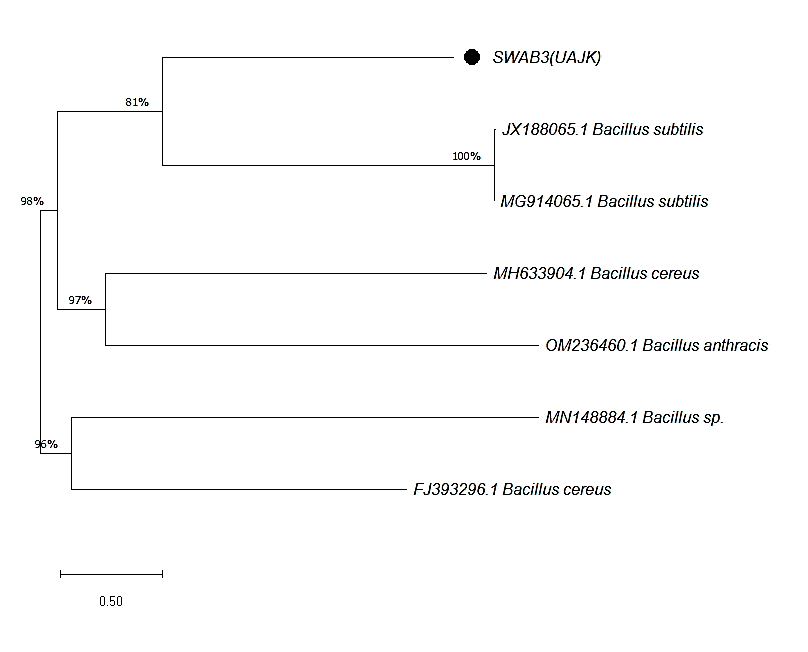
**

**Supplementary Fig 5. Phylogenetic relationship of spring water-associated bacteria SWAB 3 with other NCBI bacterial species.**

**
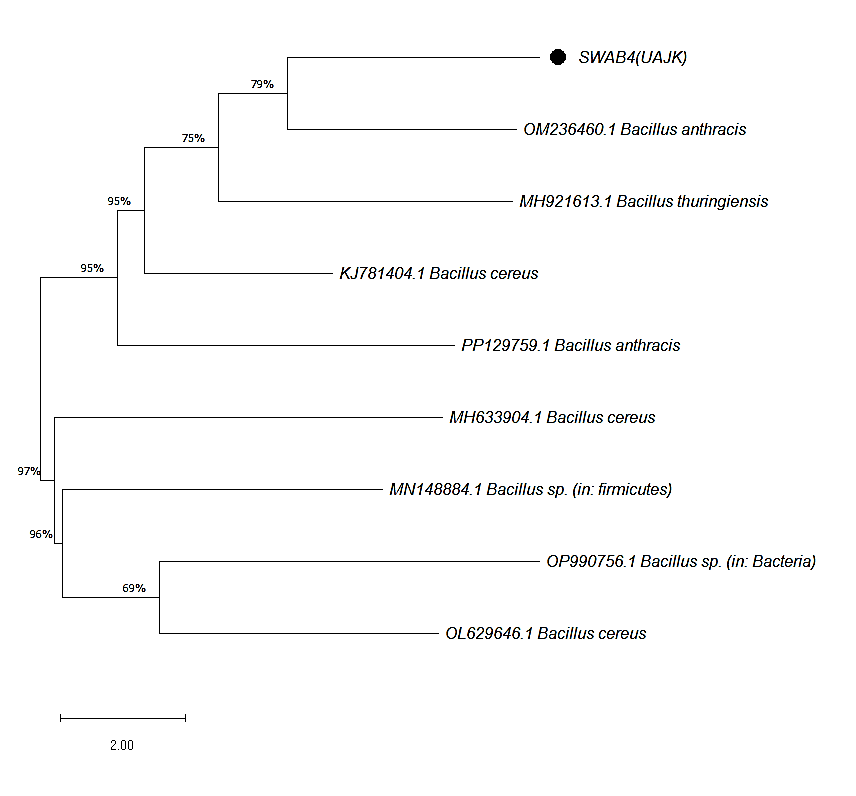
**

**Supplementary Fig 6. Phylogenetic relationship of spring water-associated bacteria SWAB 4 with other NCBI bacterial species.**

**
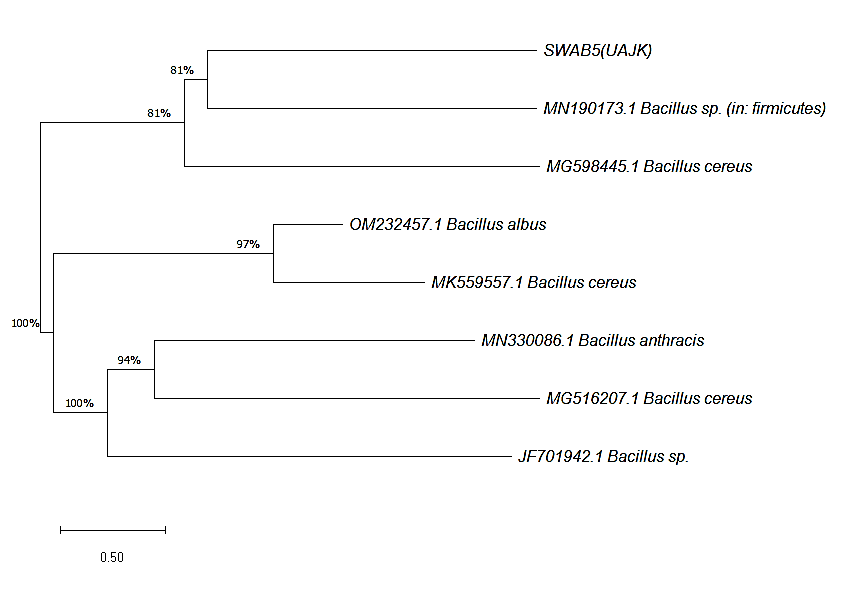
**

**Supplementary Fig 7. Phylogenetic relationship of spring water-associated bacteria SWAB 5 with other NCBI bacterial species.**

**
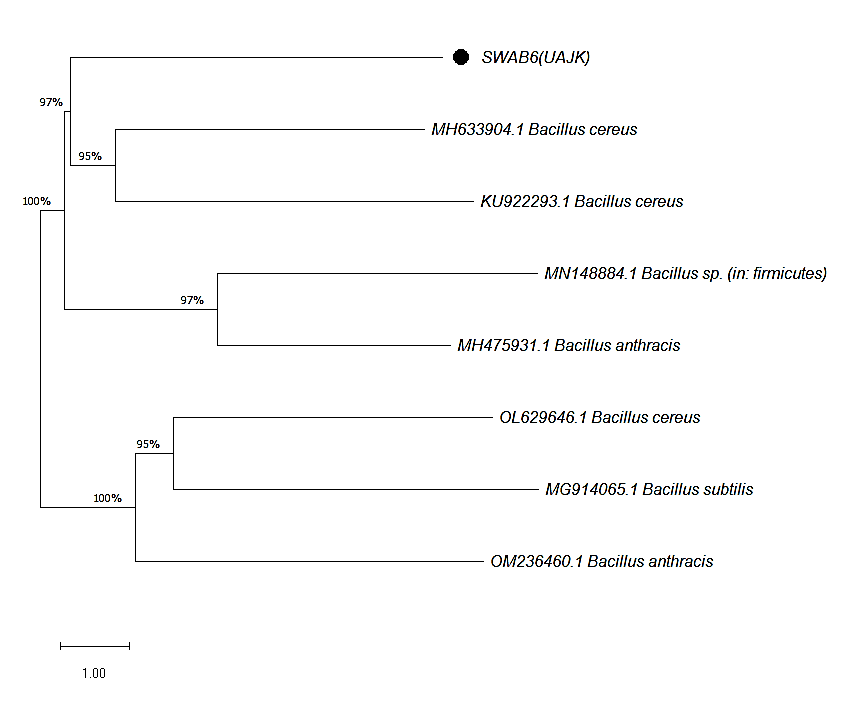
**

**Supplementary Fig 8. Phylogenetic relationship of spring water-associated bacteria SWAB 6 with other NCBI bacterial species.**

**
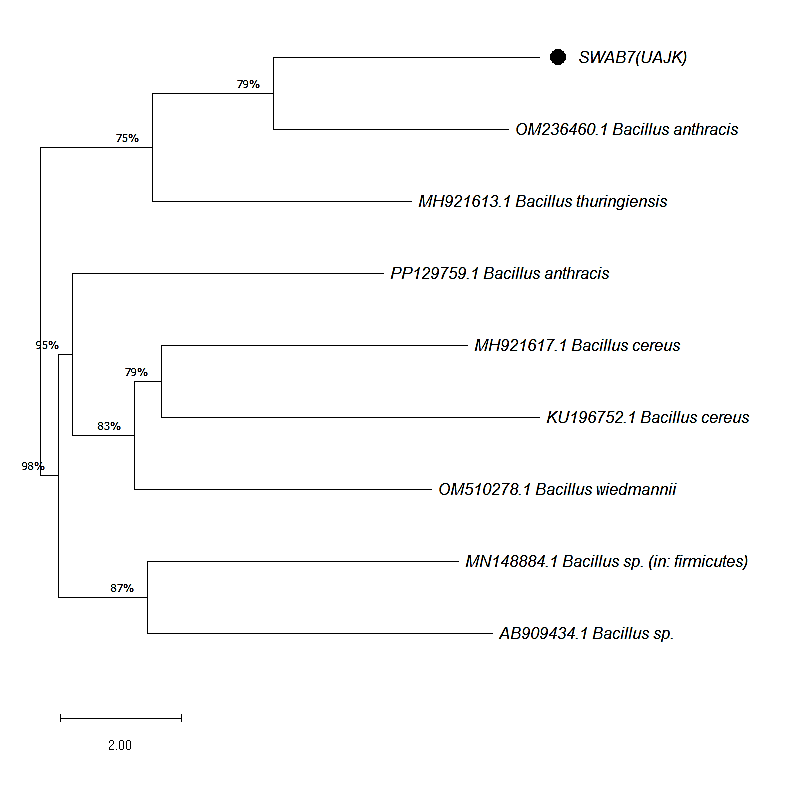
**

**Supplementary Fig 9. Phylogenetic relationship of spring water-associated bacteria SWAB 7 with other NCBI bacterial species.**

**
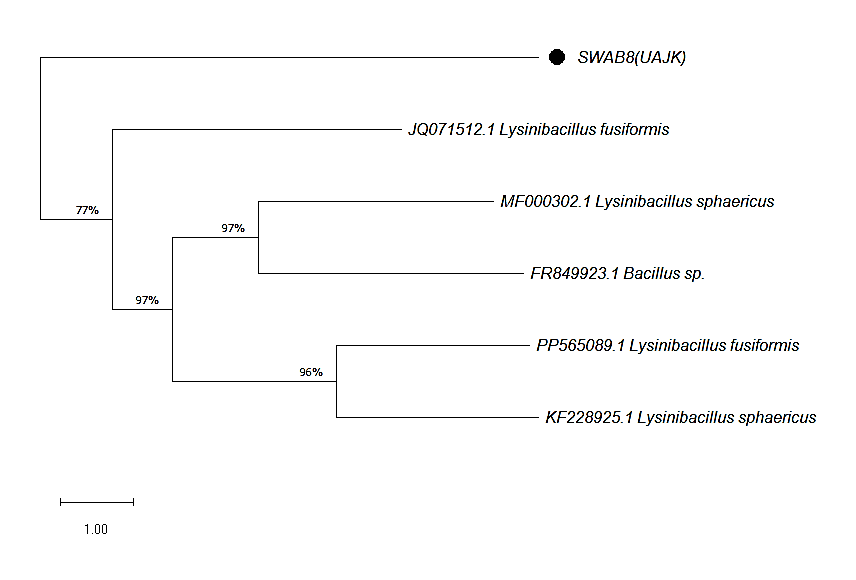
 Supplementary Fig 10. Phylogenetic relationship of spring water-associated bacteria SWAB 8 with other NCBI bacterial species.**

**
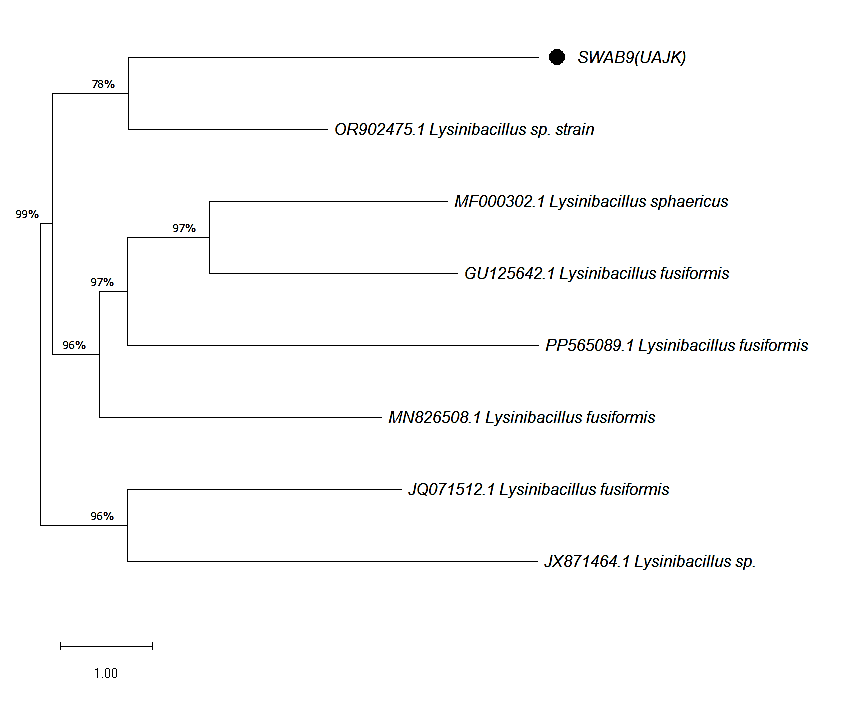
**

**Supplementary Fig 11. Phylogenetic relationship of spring water-associated bacteria SWAB 9 with other NCBI bacterial species.**

**
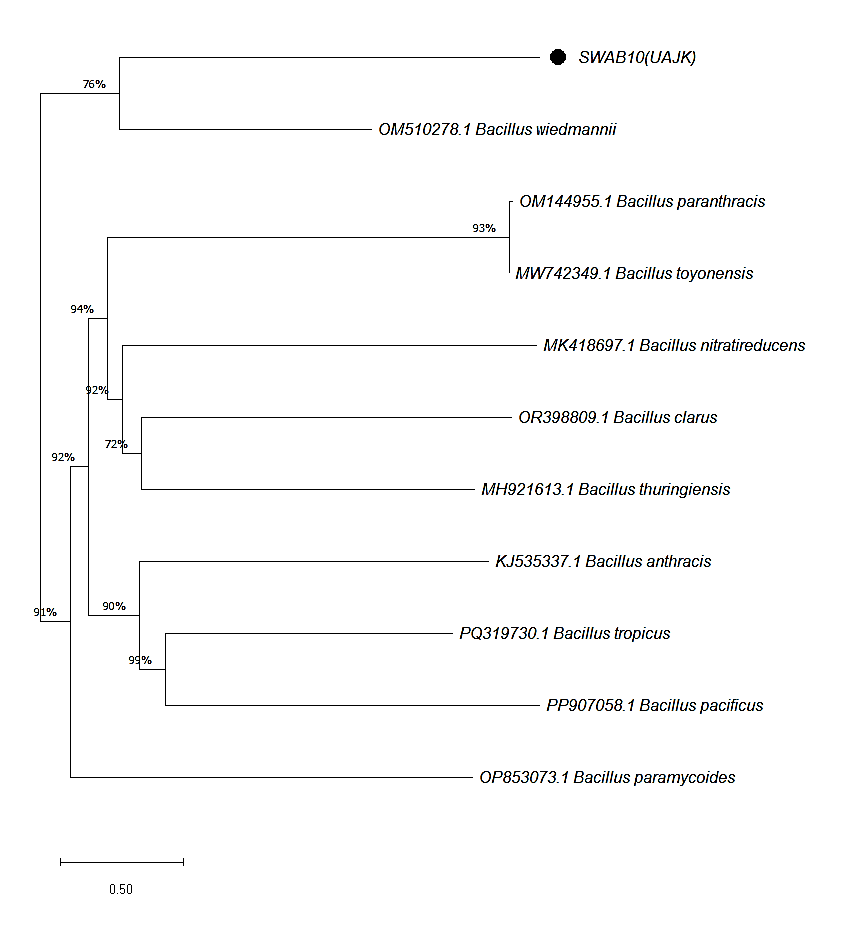
**

**Supplementary Fig 12. Phylogenetic relationship of spring water-associated bacteria SWAB 10 with other NCBI bacterial species.**

**
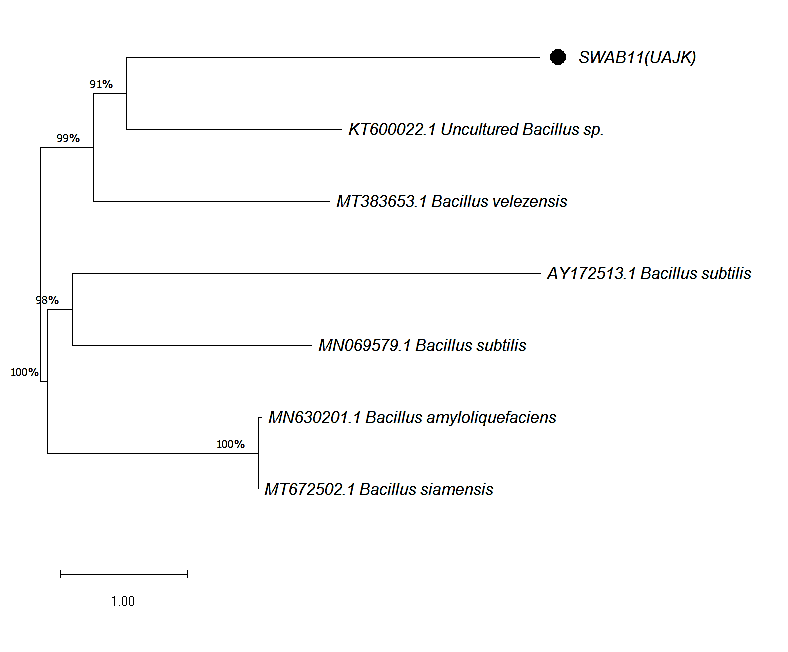
**

**Supplementary Fig 13. Phylogenetic relationship of spring water-associated bacteria SWAB 11 with other NCBI bacterial species.**

**
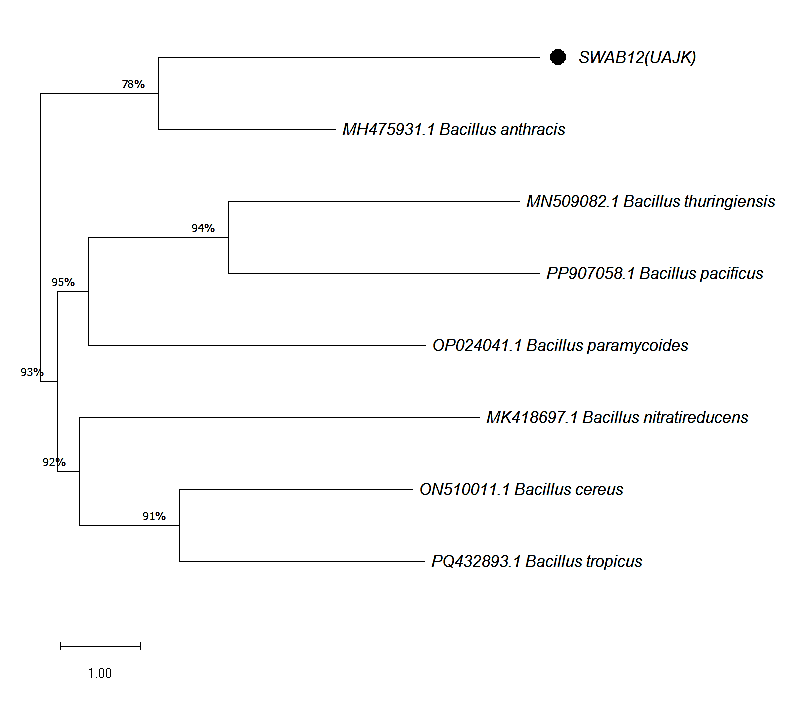
**

**Supplementary Fig 14. Phylogenetic relationship of spring water-associated bacteria SWAB 12 with other NCBI bacterial species.**

**
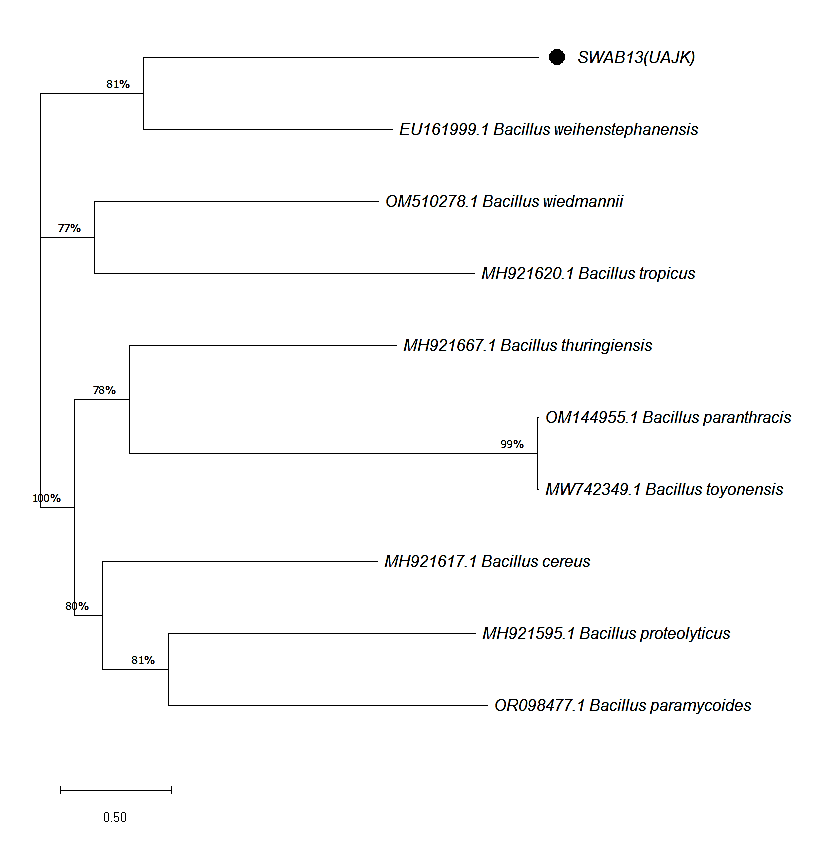
**

**Supplementary Fig 15. Phylogenetic relationship of spring water-associated bacteria SWAB 13 with other NCBI bacterial species.**

**
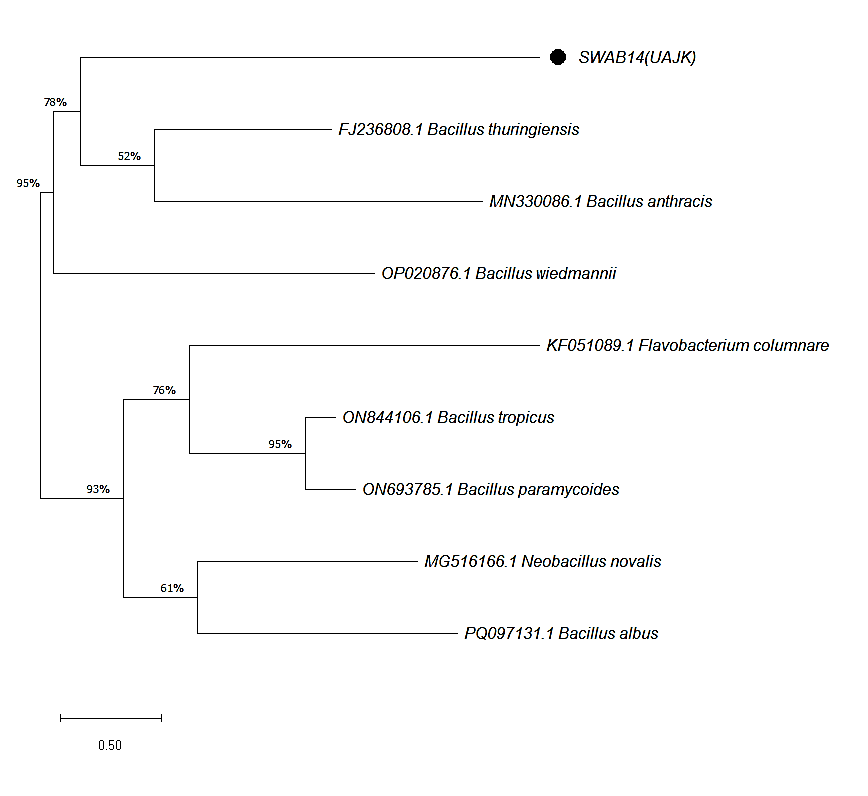
**

**Supplementary Fig 16. Phylogenetic relationship of spring water-associated bacteria SWAB 14 with other NCBI bacterial species.**

**
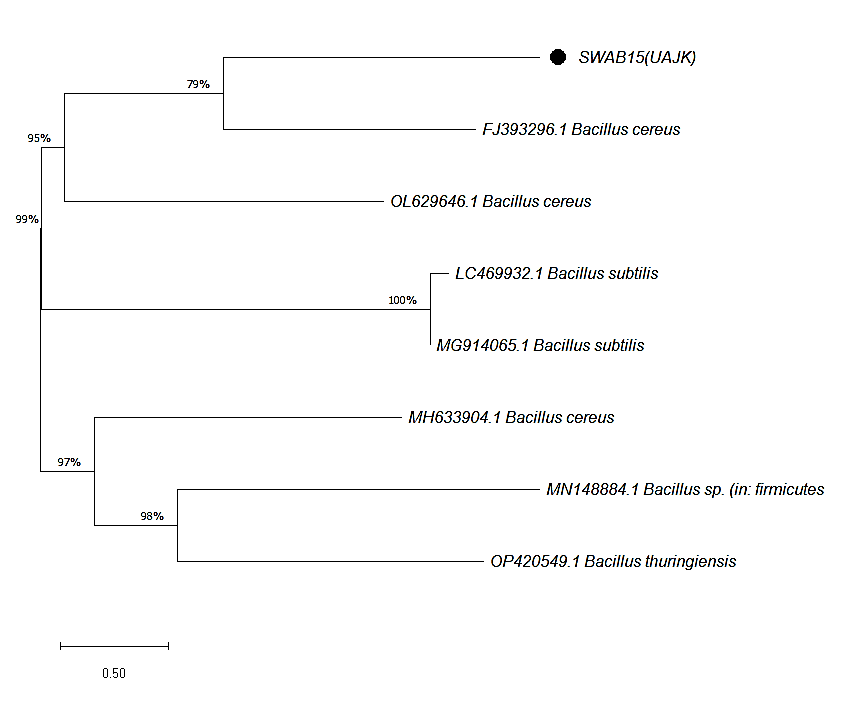
**

**Supplementary Fig 17. Phylogenetic relationship of spring water-associated bacteria SWAB 15 with other NCBI bacterial species.**


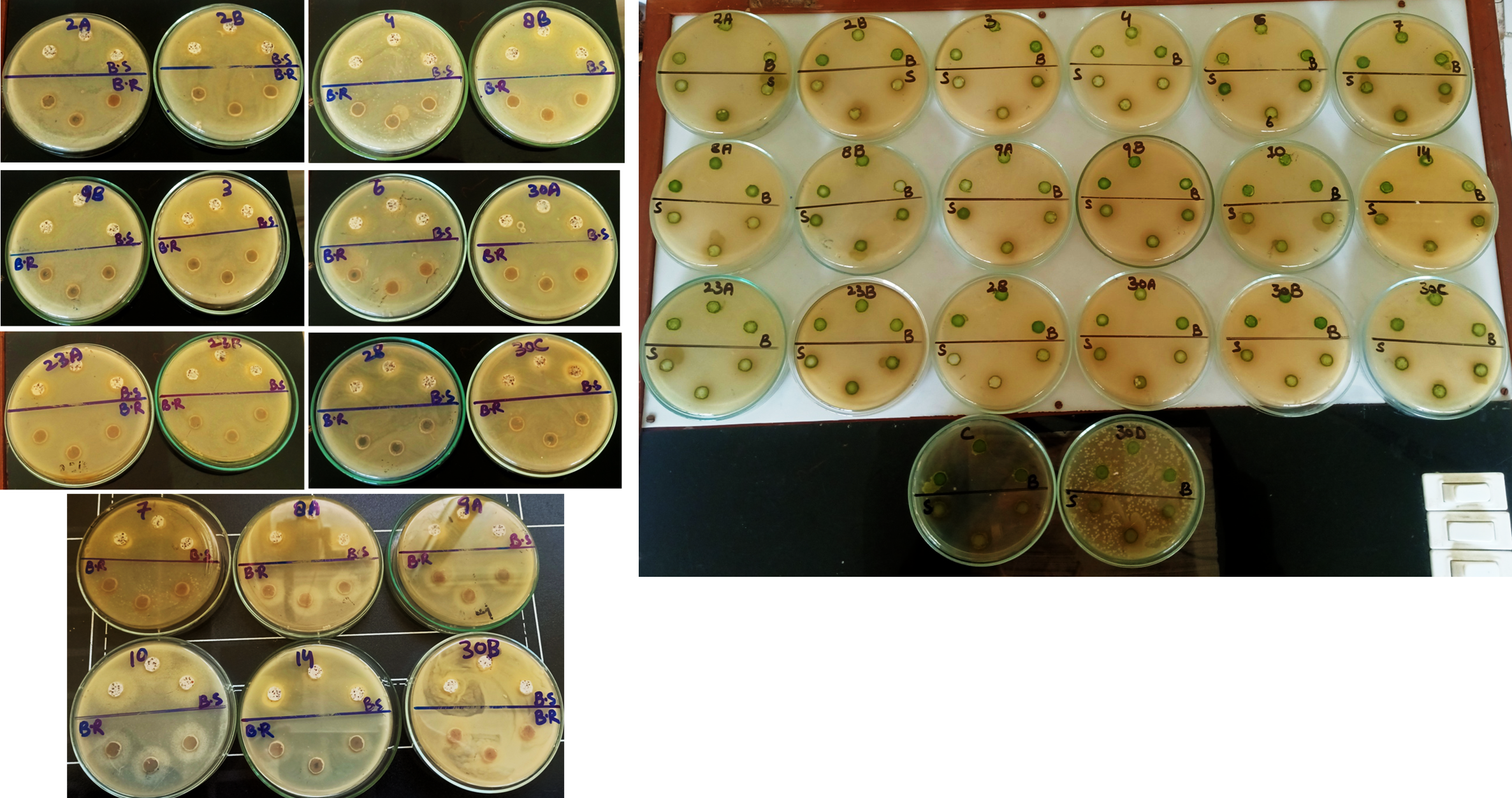


**Supplementary Fig 18. Antibacterial efficacy of B. rapa and *S. oleracea* on spring water associated bacteria**
